# CEEMDAN–SST-GraphPINN-TimesFM Model Integrating Operating-State Segmentation and Feature Selection for Interpretable Prediction of Gas Concentration in Coal Mines

**DOI:** 10.3390/s26082476

**Published:** 2026-04-17

**Authors:** Linyu Yuan

**Affiliations:** College of Safety Science and Technology, Xi’an University of Science and Technology, Xi’an 710064, China; 23403070332@stu.xust.edu.cn

**Keywords:** CEEMDAN–SST, GraphPINN, TimesFM, early-warning model, interpretability

## Abstract

Gas concentration series in coal mining faces are jointly affected by multiple coupled factors, including geological conditions, mining disturbances, ventilation organization, and gas drainage intensity, and therefore exhibit pronounced nonstationarity, strong fluctuations, spatiotemporal correlations across multiple monitoring points, and occasional abrupt spikes. To address these challenges, this study proposes a gas concentration prediction and early-warning method that integrates CEEMDAN–SST with GraphPINN-TimesFM (Graph Physics-Informed Neural Network–Time Series Foundation Model). First, based on multi-source monitoring data such as wind speed, gas concentrations at multiple monitoring points, and equipment operating status, anomaly removal, operating-condition segmentation, and change-point detection are performed to construct stable operating-state labels. Feature selection is then conducted by combining optimal time-lag correlation, Shapley value contribution, and dynamic time warping. Second, WGAN-GP is employed to augment samples from minority operating conditions, while CEEMDAN–SST is used to decompose and reconstruct the target series so as to reduce the interference of nonstationary noise and enhance sequence predictability. On this basis, TimesFM is adopted as the backbone for long-sequence forecasting to capture long-term dependency features in gas concentration evolution. Furthermore, GraphPINN is introduced to embed the topological associations among monitoring points, airflow transmission delays, and convection–diffusion mechanisms into the training process, thereby enabling collaborative modeling that integrates data-driven learning with physical constraints. Finally, the predictive performance, early-warning capability, and interpretability of the proposed model are systematically evaluated through regression forecasting, warning discrimination, and Shapley-based interpretability analysis. The results demonstrate that the proposed method can effectively improve the accuracy, robustness, and physical consistency of gas concentration prediction under complex operating conditions, thereby providing a new technical pathway for gas over-limit early warning and safety regulation in coal mining faces.

## 1. Introduction

Gas is one of the major hazardous factors in coal mining faces. Its emission and migration are jointly governed by multiple factors, including seam occurrence conditions, mining-induced disturbance, pressure relief in surrounding rock, ventilation organization, gas drainage intensity, and production rhythm, thereby exhibiting pronounced nonlinearity, nonstationarity, and abrupt variability. Owing to the layout of underground sensors and the constraints of field data acquisition, on-site monitoring data are generally recorded in the form of continuous time series, most of which consist of multivariate coupled sequences involving wind speed, gas concentrations at multiple monitoring points, and other operating-condition-related variables. Such data are characterized not only by trend drift, local spikes, and strong noise interference, but also by significant spatial correlations and transmission delays among different monitoring points. Therefore, achieving highly accurate, robust, and interpretable prediction of gas concentration under complex operating conditions has become a central issue in mine safety monitoring and hazard early warning.

Considerable efforts have been made by researchers worldwide to address coal mine gas concentration prediction. Early studies mainly relied on shallow learning methods, such as artificial neural networks, for nonlinear fitting. Karacan et al. [1] employed a supervised artificial neural network to model methane emissions from mine ventilation systems under longwall mining conditions, demonstrating the learnability of the complex nonlinear relationship between production parameters and methane emissions. Ślęzak et al. [2] developed a multisensor predictive framework for coal mine decision support systems and pointed out that the fusion of multisource monitoring data can enhance the engineering applicability of methane concentration prediction. With the rapid development of deep learning, time-series modeling approaches based on recurrent neural networks have gradually become a major focus in this field. Zhang et al. [3] established an LSTM prediction model based on multivariate time series and verified its effectiveness in capturing short-term dependency patterns in gas concentration data. Lyu et al. [4] further combined an encoder–decoder architecture with LSTM, thereby improving robustness in scenarios involving multisensor inputs and multi-step forecasting.

To address the strong noise, severe fluctuations, and pronounced multifactor coupling in underground gas concentration series, many studies have attempted to improve model performance through feature enhancement and architectural refinement. Song et al. [5] proposed a multi-parameter fusion RNN model for the dynamic prediction of pressure relief gas concentration, showing that joint modeling of environmental and production variables can improve predictive performance under complex operating conditions. Dey et al. [6] integrated t-SNE, variational autoencoders, and Bi-LSTM for gas concentration prediction in underground sealed areas, thereby strengthening the model’s ability to represent complex nonlinear features. Meng et al. [7] combined deep learning methods with classical time-series analysis, improving both the accuracy and fitting stability of short-term methane concentration prediction. These studies indicate that data-driven approaches centered on recurrent neural networks have achieved encouraging progress in gas concentration forecasting, although their performance still depends heavily on sample quality and model architecture design.

With the continuous improvement of underground multi-point monitoring systems, spatiotemporal correlation modeling has gradually become an important direction for enhancing gas concentration prediction. Cheng et al. [8] proposed a temporal graph convolutional network enhanced by evolutionary attention to learn gas propagation and coupling relationships among multiple monitoring points. Wen et al. [9] developed a deep learning-based prediction and early-warning model for fully mechanized mining faces, showing that the joint characterization of environmental factors and production states is crucial for improving warning reliability. Diaz et al. [10] compared the performance of univariate and multivariate models for underground methane prediction and found that multivariate modeling is generally more advantageous under complex operating conditions. Gao et al. [11] further proposed an attention-enhanced spatiotemporal model and verified that the joint representation of spatial topological relationships and temporal dependencies contributes significantly to methane concentration prediction accuracy. These findings suggest that the evolution of gas concentration is not only strongly time-dependent but also profoundly shaped by spatial propagation mechanisms, making it difficult for purely temporal fitting methods to fully characterize its dynamic behavior.

On the other hand, denoising, decomposition, and reconstruction methods have also been widely introduced to address the strong nonstationarity and multiscale disturbances of gas time series. Gao et al. [12] combined wavelet threshold denoising, phase-space reconstruction, and LSTM, which suppressed noise interference while enhancing the predictable structure of the series. Tutak et al. [13] demonstrated the feasibility of deploying multilayer perceptrons for underground methane concentration prediction, but also pointed out that lightweight models often suffer from insufficient generalization under operating-condition drift and abrupt change scenarios. In recent years, models targeting long-sequence dependencies and complex-scene forecasting have continued to emerge. Liu et al. [14] achieved integrated prediction and early warning based on the MTGNN-Bayesian-IF-DBSCAN algorithm. Yuan [15] proposed a decomposition-enhanced cross-graph prediction and fluctuation monitoring method to improve both time-series forecasting and anomaly identification. Chang et al. [16] combined explainable artificial intelligence with advanced feature selection methods to identify the key driving factors of gas concentration in longwall faces, highlighting the value of interpretability analysis in safety early warning. Xu [17] developed a GCN-Crossformer model, further strengthening the joint representation of spatial topological relationships and long-range dependency features. Liu et al. [18] conducted multi-information fusion forecasting based on Informer, indicating that Transformer-based architectures hold considerable promise for long-sequence modeling of coal mine gas data.

Although existing studies have significantly advanced coal mine gas concentration prediction from empirical judgment toward data-driven analysis, several key challenges remain unresolved. First, most methods rely primarily on statistical correlations for fitting and have not explicitly incorporated physical mechanisms such as airflow driving, spatial propagation, convection–diffusion, and source sink variation into the modeling framework. As a result, they are prone to producing physically inconsistent predictions under trend drift, extreme spikes, and cross-condition extrapolation scenarios. Second, existing methods are often highly sensitive to sample partitioning, hyperparameter settings, and stochastic training processes, making stable and reproducible model performance difficult to achieve. Third, current interpretability analyses are usually established on a preselected optimal model and are therefore susceptible to model selection bias, which limits their ability to comprehensively reveal the intrinsic relationship between prediction outcomes and physical constraints.

To address these issues, physics-informed networks provide a new avenue for modeling complex industrial processes. Raissi et al. [19] proposed Physics-Informed Neural Networks (PINNs), which unify data fitting and physical constraints by incorporating partial differential equation residuals into the loss function. For convection–diffusion–reaction problems, Hou et al. [20] demonstrated the effectiveness of PINNs in solving ADR equations. Huang et al. [21] applied PINNs to the modeling of convection–diffusion-Langmuir adsorption processes, further confirming the positive role of physical residual constraints in improving model stability and physical consistency. Meanwhile, the advantages of Transformers in modeling long-range dependencies have also opened new possibilities for predicting complex spatiotemporal processes. Boya and Subramani [22] proposed PINTO, which combines Transformer neural operators with physical constraints, offering useful inspiration for unified modeling based on “attention mechanisms + physical law embedding.” These developments suggest that integrating Transformer-like architectures with strong long-sequence modeling capability and PINN-based methods with physical consistency constraints may represent an important future direction for coal mine gas concentration prediction.

Motivated by this, this study develops a gas concentration prediction and early-warning framework that couples CEEMDAN-SST with GraphPINN-TimesFM (Graph Physics-Informed Neural Network–Time Series Foundation Model) for multisource monitoring data from coal mining faces, including wind speed, gas concentrations at multiple monitoring points, and equipment operating status. First, stable operating-condition labels are constructed through anomaly removal, operating-condition segmentation, and change-point detection, while feature selection is completed by combining optimal time-lag correlation, Shapley value contribution, and dynamic time warping. Second, WGAN-GP is employed to augment samples under minority operating conditions, and CEEMDAN-SST is used to decompose and reconstruct the target series so as to mitigate the influence of nonstationary noise. On this basis, TimesFM is adopted as the backbone for long-sequence forecasting, and GraphPINN is introduced to characterize the topological associations among monitoring points, airflow transmission delays, and convection–diffusion physical mechanisms, thereby enabling collaborative modeling driven by both data and physics. Finally, model performance is systematically evaluated from multiple dimensions, including driving factors, prediction errors, and physical dependencies, through regression assessment, early-warning discrimination, and Shapley-based interpretability analysis. This study is expected to provide more stable, reliable, and interpretable technical support for accurate gas concentration prediction, over-limit warning, and safety regulation in fully mechanized mining faces.

## 2. Materials and Methods

### 2.1. Data Acquisition

**Figure 1:**
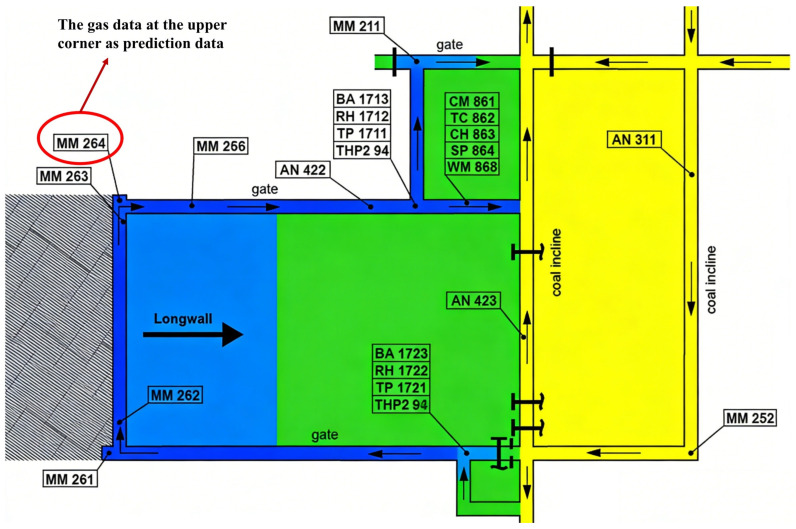
Sensor distribution and layout.

**Figure 2:**
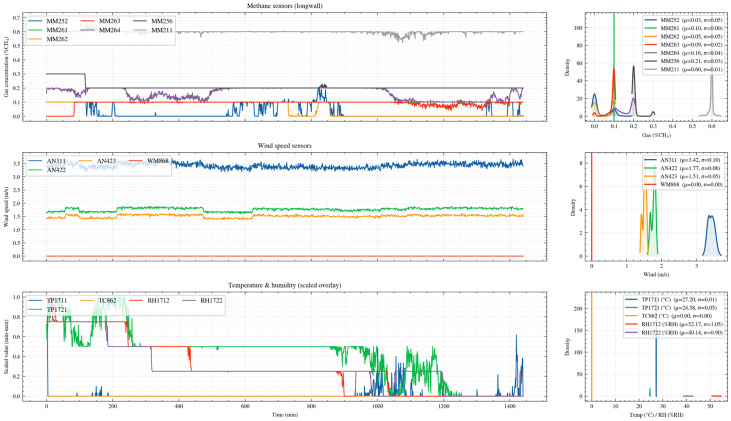
Distribution of sensor data.

The data used in this study were obtained from the monitoring system of a longwall mining face in the Upper Silesian Coal Basin, Poland, covering the period from March 2014 to 16 June 2014 [23]. The dataset includes key environmental variables such as methane concentration, air velocity, temperature, humidity, and air pressure, together with the operating-state signals of coal mining equipment, thereby forming a high-frequency multidimensional time-series dataset. Specifically, the dataset contains synchronized observations from two sensor arrays spanning from 2 March 2014, to 16 June 2014, comprising a total of 9,199,930 records. Each record consists of a timestamp and measurements from 28 sensor channels. Figure 1 illustrates the layout of the longwall face and the roadway system, where fresh airflow enters through the intake roadway, passes along the mining face, and is discharged through the return airway. Figure 2 presents the overall variation trend of methane concentration. The sequence exhibits pronounced temporal dependence and multivariate co-evolution characteristics, while variables such as air velocity, temperature, and humidity also display consistent temporal patterns.

The sensor deployment consists of two types of systems, which were synchronized through a central dispatching platform. One category includes underground environmental monitoring sensors, such as methane, air velocity, air pressure, temperature, and humidity probes. These sensors were mainly installed along the longwall face and at key locations in the intake and return roadways to cover gas-prone accumulation zones, with data collected by the SMPNT safety system. The other category includes shearer operating-state sensors, which record equipment conditions such as current, traction speed, and travel direction, with data acquired by the MAKS DBC system. Signals from both systems were temporally aligned and integrated by the THOR dispatching system, forming a unified high-frequency monitoring dataset that jointly captures environmental conditions and production states.

Given that the upper corner region is particularly susceptible to local gas accumulation due to airflow recirculation and gas emission from the goaf, which increases underground safety risk, this study focuses on the methane concentration measured at monitoring point MM264 near the upper corner. A prediction model for future time steps is then established to characterize and provide early warning for the dynamic fluctuation process of gas concentration in this critical area.

### 2.2. Data Preprocessing Methods

#### 2.2.1. Operating-State Segmentation Method

Based on the existing dataset, data preprocessing was first carried out. Specifically, outlier removal was performed by calculating the quartiles and the interquartile range (IQR) for each variable x, and unreasonable values were removed to reduce the interference of extreme spikes in subsequent modeling. The outlier removal criterion adopted in this study was 1.5×IQR.

On this basis, the complex production process was discretized into an interpretable state sequence. An activity index was constructed, and the Otsu adaptive thresholding method [24] was employed to identify operating states such as production, coal cutting, and movement. A z-score was then constructed for each sensor x, as shown in Equation (1):

(1) $$z=\frac{x-\mathrm{median}(x)}{1.4826\cdot \mathrm{MAD}\left(x\right)+\varepsilon },\;\mathrm{MAD}(x)=\mathrm{median}(|x-\mathrm{median}(x)|)$$

where $x_{t}^{j}$ denotes the raw observation of the j-th sensor at time t, with units corresponding to the physical unit of that sensor. Its value range is constrained by the sensor measurement scale, and the data are obtained from raw acquisition after outlier and invalid-value removal. $\mathrm{median}(x^{j})$ denotes the median of sequence $x^{j}$, with the same unit as $x_{t}^{j}$; it is recommended to compute it column-wise within the training period to avoid information leakage. MAD(x) denotes the median absolute deviation, defined as median(∣x−median(x)∣), with the same unit as $x_{t}^{j}$, and its value is nonnegative. The constant 1.4826 is used to convert the MAD into a scale consistent with the standard deviation under approximate normality. ε is a positive numerical stability term, set to $10^{-12}$, to avoid division by zero.

Subsequently, a set of activity-related sensors $S_{act}$, such as AMP, DMP, and speed, was defined, and the activity index $A_{t}$ was constructed as follows:

(2) $$A_{t}=\sum_{j\in S_{act}}\;\left|z_{t}^{j}\right|$$

where $A_{t}$ is the activity index, which is dimensionless and takes nonnegative real values. A larger value indicates a higher intensity of production activity. $S_{act}$ denotes the set of activity-related sensors and may be determined based on signals such as shearer current, traction speed, travel direction, and other variables reflecting operating states.

Thereafter, Otsu’s method was used to determine the threshold that maximizes the between-class variance, as shown in Equation (3):

(3) $$\theta^{*}=arg\;\underset{\theta \in \Theta }{max}\;\sigma_{B}^{2}(\theta ),\sigma_{B}^{2}(\theta )=\omega_{0}(\theta )\omega_{1}(\theta ){\left[\mu_{0}(\theta )-\mu_{1}(\theta )\right]}^{2}$$

In Equation (3), $\theta^{*}$ denotes the production discrimination threshold, which is dimensionless and has the same scale as $A_{t}$. Θ is the candidate threshold set, consisting of the bin centers of the histogram of $A_{t}$. $\omega_{0}(\theta )$ and $\omega_{1}(\theta )$ represent the sample proportions on the two sides of the threshold, respectively, both ranging from 0 to 1 and summing to 1. $\mu_{0}(\theta )$ and $\mu_{1}\left(\theta \right)$ denote the mean values of the samples on the two sides of the threshold and have the same scale as $A_{t}$.

Next, state encoding and minimum-duration constraints were designed. Taking production and coal-cutting states as examples, the corresponding state indicators were defined as follows:

(4) $$y_{t}^{prod}=I(A_{t}\geq \theta^{*})$$

(5) $$C_{t}=\sum_{j\in S_{cut}}\;\left|z_{t}^{j}\right|,y_{t}^{cut}=y_{t}^{prod}\cdot I(C_{t}\geq \theta_{cut})$$

In Equation (4), $y_{t}^{prod}\in \{0,1\}$ denotes the production-state indicator, where $y_{t}^{prod}=1$ indicates that the system is in the production state. I(⋅) is the indicator function, which equals 1 when the condition is satisfied and 0 otherwise. In this study, the activity index $A_{t}$ was constructed from the robustly standardized signals $\left\{AMP1\_IR,AMP2\_IR,DMP3\_IR,DMP4\_IR,AMP5\_IR,V\right\}$, and the production threshold $\theta^{*}$ was determined by Otsu’s method. In Equation (5), $C_{t}$ denotes the coal-cutting intensity indicator. In this study, $S_{cut}$ was defined as $\left\{AMP1\_IR,AMP2\_IR\right\}$, and $C_{t}$ was taken as the maximum of these two cutting-related signals at time t after temporal smoothing. The threshold $\theta_{cut}$ was estimated by Otsu’s method using production-period samples only and was then kept fixed during the test period to avoid information leakage. Since coal cutting is a subset of active production, $y_{t}^{cut}=1$ indicates a coal-cutting state within the production phase, whereas $y_{t}^{prod}=1$ and $y_{t}^{cut}=0$ indicate production without coal cutting.

The final state $state_{t}$ is defined in Equation (6). Segments shorter than a specified threshold are merged and corrected to improve the stability of the state sequence. Here, state=1 indicates production without coal cutting, whereas state=2 indicates coal cutting:

(6) $${state}_{t}=\left\{\begin{array}{c} 0,\;y_{t}^{prod}=0\\ 1,\;y_{t}^{prod}=1\\ 2,\;y_{t}^{cut}=1 \end{array}\right.\wedge y_{t}^{cut}=0$$

where $state_{t}$ is the discrete operating-condition label. To suppress jittery switching, segments whose continuous duration is shorter than the minimum-duration threshold should be merged and corrected. The minimum-duration threshold may be expressed in units of time or number of steps and should be determined according to the sampling interval and the typical duration of state transitions in the field, so as to improve label stability by reducing frequent short-term switching.

#### 2.2.2. Change-Point Detection Method

After constructing the operating-state segmentation method, a key control signal s(t) was smoothed using a rolling median, and a lagged difference magnitude was constructed as follows:

(7) $${\tilde{s}}_{t}=med\left(s_{t-w+1},\ldots ,s_{t}\right),\;\Delta_{t}=\left|{\tilde{s}}_{t}-{\tilde{s}}_{t-l}\right|$$

where $s_{t}$ denotes the control signal used for change-point detection, such as ventilation flow or a gas-drainage-related signal, with units corresponding to its physical meaning. ${\tilde{s}}_{t}$ denotes the rolling median smoothing result with window length w, having the same unit as $s_{t}$. w is the smoothing window length, expressed in steps or time units, and should be selected according to the sampling interval and noise level. l is the lag step, expressed in number of steps, and is typically chosen as one or several steps to highlight abrupt changes. $\Delta_{t}$ is the difference magnitude and is nonnegative.

An MAD-based robust thresholding method was then used to determine the stable threshold and the set of candidate change points, as shown in Equation (8):

(8) $$\eta =\mathrm{med}(\Delta )+\kappa \cdot 1.4826\cdot \mathrm{MAD}(\Delta ),\;t\in C\Leftrightarrow \Delta_{t}\geq \eta$$

In Equation (8), η denotes the robust threshold, having the same unit as $\Delta_{t}$. κ is the sensitivity coefficient, which is dimensionless and set to 4; in practice, it is usually selected from the range of 2 to 6 to balance false alarms and missed detections. C denotes the set of candidate change points. Minimum-spacing and persistence constraints may then be further introduced to avoid dense noise-triggered detections.

To prevent test-set leakage, all quantities involving statistical estimation during computation, including median(⋅), MAD(⋅), $\theta^{*}$, $\theta_{cut}$, and threshold η, were calculated exclusively within the training period and kept fixed during the test period without further updating.

**Figure 3:**
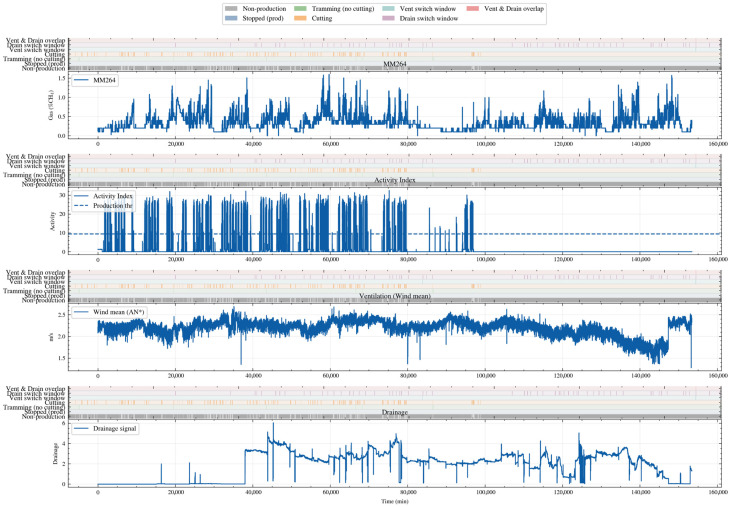
Results of operating-condition classification.

The screening results are shown in Figure 3. From top to bottom, the figure presents the MM264 concentration sequence and the Activity Index obtained by summing multiple activity-related signals after Robust Z-score standardization. The dashed line indicates the production discrimination threshold determined by the Otsu adaptive method, which is used to distinguish production from non-production states. The mean ventilation or air-velocity signal and the drainage signal are then shown separately. Colored bars at the top of each subplot denote the operating-condition labels: gray indicates non-production, blue indicates production but shutdown, green indicates movement without coal cutting, and orange indicates coal cutting. In addition, the figure overlays the ventilation switching windows, drainage switching windows, and their overlapping intervals identified by the robust change-point detection method. The results show that significant fluctuations and peaks in MM264 concentration are mainly concentrated in production stages where the Activity Index exceeds the threshold, and they are more pronounced under coal-cutting conditions and some movement conditions. The switching windows of ventilation and drainage are highly consistent with the step changes and short-term disturbances in the wind mean or drainage signals, and overlapping windows of the two types can also be observed during a few time intervals.

### 2.3. Feature Engineering and Analysis Method

**Figure 4:**
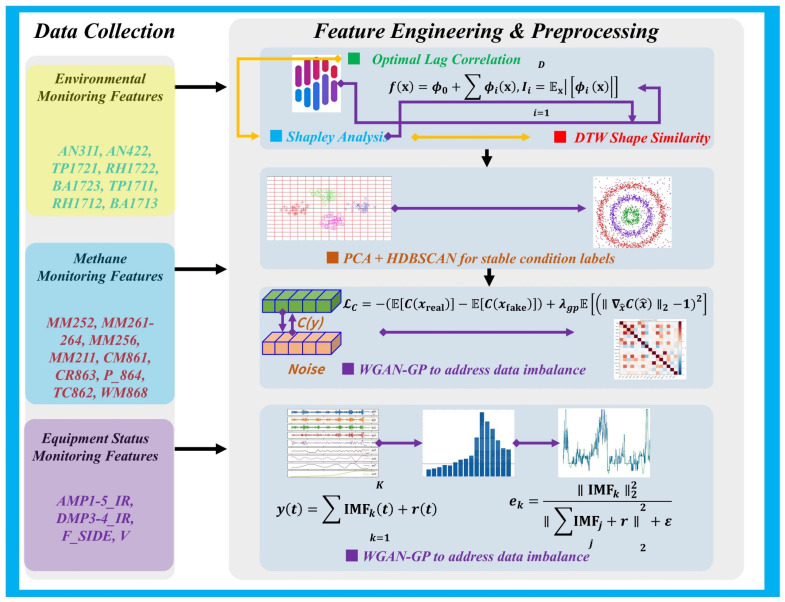
Flowchart of feature selection and operating-condition analysis.

After completing the above procedures, feature engineering and data preprocessing analysis were carried out. The overall framework is shown in Figure 4, which summarizes the end-to-end process from multisource monitoring data to the representations and sample sets used for modeling. The left side of the figure presents the acquisition and aggregation of three categories of sensor variables, namely environmental monitoring, methane monitoring, and equipment-state monitoring, while the right side shows the key steps of feature engineering and preprocessing. Wind mean (AN*) represents the ventilation representative value obtained by averaging the signals of all AN series wind speed sensors.

First, based on the training data, the association strength between candidate variables and the target sequence was characterized from three perspectives: optimal lagged correlation, contribution assessment based on Shapley values, and morphological similarity measured by dynamic time warping. These multiple sources of evidence were then normalized and fused to screen out a compact and interpretable subset of key features.

To avoid data leakage, all feature-selection and preprocessing operations were performed in a training-only manner. Specifically, the lagged-correlation analysis, SHAP-based contribution estimation, and DTW-based similarity evaluation were computed exclusively on the training set. The resulting feature subset and associated lag settings were then fixed and applied to the test set without refitting. Likewise, all statistical quantities required in preprocessing, including missing-value handling rules, scaling parameters, PCA fitting, HDBSCAN clustering, and reconstruction-related criteria, were estimated from the training data only and kept unchanged during test-time transformation and evaluation.

Next, production-segment samples were standardized, and principal component analysis was performed to extract low-dimensional structural representations. HDBSCAN [25] density clustering was then employed to obtain stable operating-condition labels, thereby providing consistent state constraints for subsequent conditional modeling and sample weighting.

To alleviate the learning bias caused by imbalanced operating-condition distributions, a Wasserstein generative adversarial network with gradient penalty [26] was further introduced to generate sequential samples, thereby augmenting minority operating conditions and improving coverage of the training distribution. Finally, CEEMDAN decomposition was applied to the target methane sequence, representing the signal as several intrinsic mode functions and a residual term. Effective modes were then selected for reconstruction based on energy proportion and correlation criteria, so as to suppress nonstationary noise while preserving key dynamic components.

### 2.4. GraphPINN-TimesFM Prediction Model with Graph–Physical Data Fusion

#### 2.4.1. Mechanistic Integration of TimesFM and GraphPINN

To explicitly characterize the interaction between long-sequence forecasting and physics-guided regularization, the proposed framework couples a TimesFM forecasting backbone with a GraphPINN correction-and-constraint branch. Let $X_{t}=\{x_{t-L+1},x_{t-L+2},\ldots ,x_{t}\}\in R^{L\times d}$ denote the multivariate input window at time t, where L is the look-back length and d is the number of input variables after feature selection, operating-state encoding, and sequence reconstruction.

The TimesFM backbone first maps the input window to a baseline long-horizon representation and produces a baseline forecast as Equation (9).

(9) $${\hat{y}}_{t}^{base}=F_{\theta }(X_{t})$$

where $F_{\theta }(\cdot )$ denotes the TimesFM backbone with parameters θ, and ${\hat{y}}_{t}^{base}$ is the baseline prediction of the reconstructed methane concentration at the target point.

In the proposed framework, GraphPINN does not replace the forecasting backbone; instead, it interacts with TimesFM through two tightly coupled pathways: a residual correction pathway and a graph–physical constraint pathway. The residual correction pathway is used to refine the baseline forecast under complex operating conditions, while the graph–physical pathway injects topology-aware transport constraints into the training objective. In this way, the final prediction is jointly influenced by the statistical long-sequence modeling capability of TimesFM and the structural regularization effect of the graph-based physical prior.

To enhance the baseline forecast, a learnable correction branch is introduced. Let $h_{t}\in R^{m}$ denote the latent forecasting representation extracted from the final hidden state of the TimesFM backbone for the prediction step at time t, and let $q_{t}=[c_{P}^{up}(t),e_{t},d_{t},s_{t},w_{t}]$ denote the auxiliary physical-context vector, where $c_{P}^{up}(t)$ is the equivalent upstream concentration, $e_{t}$ is the emission-related driving term, $d_{t}$ is the discharge-related driving term, $s_{t}$ is the operating-state label, and $w_{t}$ is the sample weight. A correction head $G_{\phi }\left(\cdot \right)$ is then used to estimate a residual term as Equation (10):

(10) $$\delta_{t}=G_{\phi }(h_{t},q_{t})$$

where ϕ  denotes the parameters of the correction branch. The corrected normalized prediction is defined as Equation (11).

(11) $${\hat{y}}_{t}^{corr}={\hat{y}}_{t}^{base}+\alpha (w_{t})\delta_{t}$$

where $\alpha (w_{t})$ is a sample-weight-dependent gated gain used to increase the correction strength for minority operating conditions or hard-to-fit intervals. After inverse normalization, the final prediction in the physical scale is written as Equation (12):

(12) $${\hat{y}}_{t}=\sigma_{y}{\hat{y}}_{t}^{corr}+\mu_{y}$$

where $\mu_{y}$ and $\sigma_{y}$ are the training-set mean and standard deviation of the prediction target, respectively.

The physical branch is constructed on the basis of a simplified first-order dynamic balance equation. Let c(t)  denote the methane concentration at the target point. Its temporal evolution is approximated as Equation (13):

(13) $$\frac{dc(t)}{dt}=a\left(t\right)\left(c^{up}\left(t\right)-c\left(t\right)\right)+b\left(t\right)e\left(t\right)-g\left(t\right)d\left(t\right)-r\left(t\right)c(t)$$

where a(t), b(t), g(t), and r(t) are non-negative time-varying physical coefficients. Here, a(t) denotes the upstream transport coupling coefficient and quantifies the airflow-driven advection effect from upstream monitoring points to the target point; b(t) is the source-emission gain coefficient and reflects the strength of methane release-related driving factors; g(t) is the discharge/removal coefficient associated with ventilation and gas drainage; and r(t) is the local decay coefficient describing the dissipation or relaxation behavior of methane concentration.

Mechanistically, the proposed GraphPINN-TimesFM framework operates through prediction-level correction and objective-level regularization rather than hard parameter sharing. TimesFM first provides a baseline long-sequence forecast that captures the dominant temporal dependency of the reconstructed methane concentration series. On this basis, a learnable correction branch further refines the baseline prediction under complex operating conditions, while the graph–physical residual constrains the corrected prediction trajectory by enforcing consistency with the simplified dynamic balance in Equation (9) and the path-aware upstream aggregation in Equation (10). In this formulation, the non-negative coefficients a, b, g, and r can be interpreted as the upstream transport coupling coefficient, source-emission gain coefficient, discharge/removal coefficient, and local decay coefficient, respectively. In implementation, these coefficients are adaptively generated from the current physical-context variables by a lightweight coefficient network and are upper-bounded to improve numerical stability. Therefore, the final model combines the long-range sequence modeling capability of TimesFM with the topology-aware physical regularization of GraphPINN, so that structurally unreasonable deviations can be suppressed during training while the forecasting backbone remains responsible for the dominant predictive signal [27].

Rather than assuming these coefficients to be fixed constants, this study models them as adaptive functions of the current system state. Specifically, a coefficient network is introduced to estimate a(t), b(t), g(t), and r(t) from the physical driver variables at time t, as shown in Equation (13). This design allows the proposed first-order dynamic equation to represent time-varying transport and source sink effects under different operating conditions, thereby improving the flexibility, stability, and physical interpretability of the model.

To improve flexibility and physical interpretability, these coefficients are not treated as fixed constants but are generated by a coefficient network as Equation (14):

(14) $$\left[a_{t},b_{t},g_{t},r_{t}]=\Psi_{\omega }(q_{t}\right)$$

where $\Psi_{\omega }(\cdot )$ is a lightweight multilayer perceptron with parameters ω. To ensure physical plausibility and numerical stability, the outputs are constrained to be non-negative, and an upper bound is imposed during training to avoid divergence.

A key feature of GraphPINN is that the upstream concentration is not represented by a single sensor only, but by a path-aware aggregation over the sensor graph. Let P denote the path-node set from the upstream region to the target location. For node k∈P, let $d_{k}$ be the path distance from node k to the target point and $u_{t}$ be the effective airflow velocity. The transport delay from node k is calculated as Equation (15):

(15) $$\tau_{k}=\frac{d_{k}}{u_{t}},\;s_{k}=round\left(\frac{\tau_{k}}{\Delta t}\right)$$

where Δt is the sampling interval. The distance-attenuation weight is defined as Equation (16):

(16) $$\xi_{k}=\frac{exp(-d_{k}/l)}{\sum_{j\in P}exp(-d_{j}/l)}$$

where l is the attenuation length scale. Accordingly, the graph-based equivalent upstream concentration is written as Equation (17):

(17) $$c_{P}^{up}(t)=\sum_{k\in P}\xi_{k}\;c_{k}(t-s_{k})$$

This formulation enables the model to account for both path topology and transport delay, thereby making the physical constraint more consistent with the actual propagation characteristics of methane concentration in a longwall ventilation environment.

Based on the above definitions, the graph–physical residual associated with the predicted concentration $\hat{c}(t)$ is expressed using a finite-difference approximation as Equation (18):

(18) $$\epsilon_{phys}(t)=\frac{\hat{c}(t+\Delta t)-\hat{c}(t)}{\Delta t}-[a_{t}(c_{P}^{up}(t)-\hat{c}(t))+b_{t}e(t)-g_{t}d(t)-r_{t}\hat{c}(t)]$$

It should be emphasized that the interaction between TimesFM and GraphPINN is realized mainly at the prediction level and the objective level, rather than through hard parameter sharing. Specifically, TimesFM is responsible for learning the dominant long-range temporal dependency, while GraphPINN refines the prediction and constrains the output trajectory through a graph-aware physical residual. Therefore, the proposed framework forms a collaborative mechanism in which the data-driven branch captures long-sequence statistical regularities and the physics-guided branch suppresses structurally unreasonable deviations.

#### 2.4.2. Total Loss Function and Training Strategy

To train the proposed model, three levels of objectives are considered: a pure data-driven objective, a standard physics-informed objective, and the proposed graph–physical objective. The data-fitting loss is defined as Equation (19):

(19) $$L_{data}=E[w_{t}({\hat{y}}_{t}-y_{t}{)}^{2}]$$

where $y_{t}$ is the ground-truth target and $w_{t}$ is the sample weight. This weighted form is used to increase the contribution of minority operating conditions and difficult intervals.

To prevent overcorrection by the residual branch, a residual regularization term is introduced as Equation (20):

(20) $$L_{\delta }=E[\delta_{t}^{2}]$$

Meanwhile, for the graph-based physical constraint, a delay-availability weight $w_{t}^{\left(d\right)}$ is introduced to reduce the influence of unavailable or weakly reliable delayed path observations. The graph–physical loss is then written as Equation (21), in addition, $L_{phys}=E[\epsilon_{phys,local}(t{)}^{2}]$:

(21) $$L_{gphys}=E[w_{t}^{\left(d\right)}\;\varepsilon_{phys}(t{)}^{2}]$$

Accordingly, the three training objectives used in this study are defined as follows:

$$L_{Base}=L_{data},\;L_{PINN}=L_{data}+\lambda_{p}L_{phys},\;L_{GraphPINN}=L_{data}+\lambda_{\delta }L_{\delta }+ramp(\tau )\lambda_{g}L_{gphys}.$$

where $\lambda_{p}$, $\lambda_{\delta }$, and $\lambda_{g}$ are loss coefficients, and ramp(τ)∈[0, 1] is a warm-up function that gradually increases the contribution of the physical term during early training epochs. This strategy is adopted because enforcing the physical residual too strongly at the beginning of optimization may destabilize the correction branch before the baseline temporal representation becomes sufficiently reliable.

The optimization variables include the parameters of the TimesFM forecasting branch θ, the correction branch ϕ, and the coefficient network ω. Their joint optimization can be written as $\underset{\theta ,\phi ,\omega }{min}L_{GraphPINN}$.

In practical training, the backbone prediction first provides a statistically strong initial forecast, after which the residual branch and the graph–physical branch iteratively refine the output. Therefore, the physical constraint takes effect in real time during long-sequence forecasting in the following sense: at each training step, the predicted sequence produced by TimesFM is immediately transformed into a physically constrained trajectory through the residual correction branch, the path-delay-based upstream aggregation, and the graph–physical loss term. This mechanism enables the model to preserve the long-range forecasting capability of TimesFM while simultaneously incorporating topology-aware transport regularization.

Finally, the complete forecasting process can be summarized as follows: (1) construct the multivariate input window and operating-state features; (2) obtain the baseline long-sequence prediction from TimesFM; (3) compute the residual correction and gated refinement; (4) build the equivalent upstream concentration from the sensor graph and transport delays; (5) estimate time-varying physical coefficients using the coefficient network; (6) compute the graph–physical residual and the integrated loss; and (7) jointly optimize the model to obtain the final physically informed prediction.

**Figure 5:**
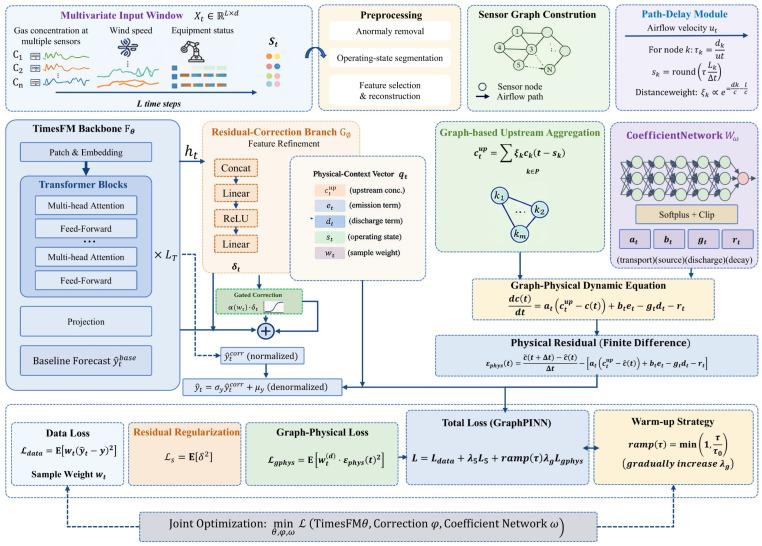
The overall modeling process and coupling mechanism of the proposed CEEMDAN–SST-GraphPINN-TimesFM framework.

Figure 5 illustrates the overall modeling process and coupling mechanism of the proposed CEEMDAN–SST-GraphPINN-TimesFM framework. First, multi-source monitoring variables form the model input within a sliding time window. After undergoing anomaly processing, operating condition division, feature selection, and sensor topology construction, they are fed into the TimesFM backbone network to extract long-sequence temporal dependencies and generate baseline predictions. On this basis, a residual correction branch is introduced to adaptively revise local deviations under complex operating conditions. Meanwhile, a path time-delay module is incorporated to perform topology-weighted aggregation of upstream monitoring point information, characterizing gas transport delays and spatial propagation effects.

Furthermore, a coefficient network dynamically estimates non-negative constraint coefficients according to the physical context, constructs a first-order graph–physical dynamic equation for the concentration evolution of target points, and forms a graph–physical constraint term through finite difference residuals. Finally, the data-fitting term, residual regularization term, and graph–physical loss term are collaboratively optimized under a warm-up strategy, enabling the model to retain the advantages of TimesFM in long-term dependency modeling while ensuring spatial topological consistency, physical rationality, and prediction stability in complex scenarios.

## 3. Results

### 3.1. Data Preprocessing and Feature Selection

The experiments were conducted on a Windows platform using Python 3.9, with an AMD 9950X3D CPU and a 5070 Ti GPU. Following the above-described methods and strategies, we first performed outlier removal on the data to prevent anomalous observations from affecting subsequent analyses.

**Figure 6:**
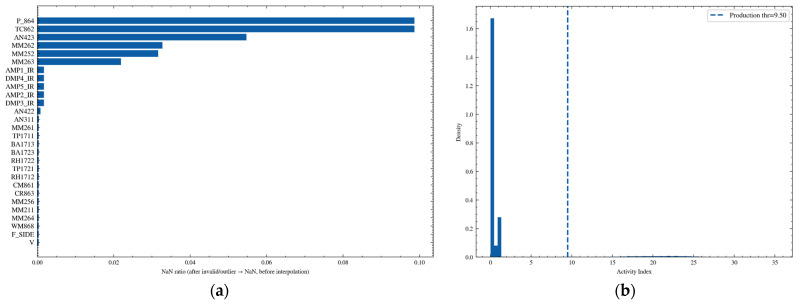
Results of sensor threshold-based filtering. (**a**) Proportion of sensor outliers removed; (**b**) Sensor classification result based on the Otsu adaptive threshold.

Figure 6 presents two key diagnostic results from the preprocessing and operating-condition threshold determination stage. Figure 6a shows the proportion of values set as missing for each sensor variable after invalid-value identification and outlier removal, which is used to evaluate the impact of data cleaning on different channels. The results indicate that a few variables, such as P_864, TC862, and AN423, exhibit relatively high missing-value ratios, suggesting that these channels experienced more frequent distortion or unavailable intervals during the observation period. Therefore, more robust missing-value handling strategies are required in subsequent feature construction to reduce interference with model inputs.

Figure 6b shows the empirical distribution of the activity index $A_{t}$. This index is obtained by applying robust standardization to multiple activity-related signals, taking their absolute values, and then summing them to characterize the intensity of production activity. The dashed line represents the production discrimination threshold determined by the Otsu adaptive thresholding method, which is 9.5. It can be observed that most samples are concentrated in the low-activity range, exhibiting a high-density distribution near zero, whereas samples exceeding the threshold correspond to production stages. This threshold was subsequently used to divide the time series into two basic states, namely production and non-production, and was further refined into operating-condition labels such as coal cutting and equipment movement, thereby providing a consistent stratification basis for subsequent change-point detection, clustering-based modeling, and weighted training.

**Figure 7:**
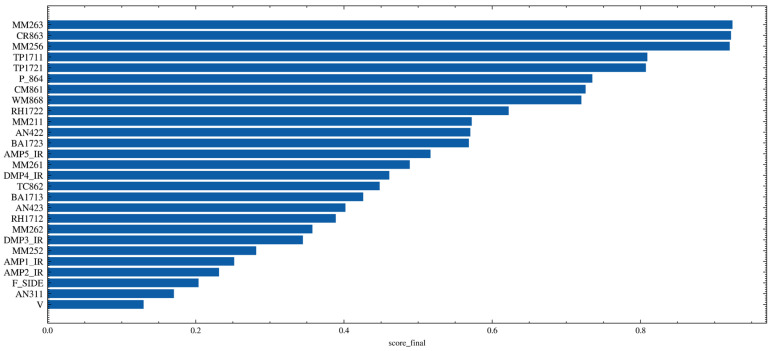
Feature ranking results.

Figure 7 shows the final ranking results of feature selection, corresponding to Table 1. The horizontal axis represents the comprehensive score, denoted as score_final, for each candidate sensor. This score is calculated from the final blended metric and integrates three types of information: lagged correlation features, model interpretability contribution, and sequence-shape similarity. The bar length is positively correlated with overall feature performance; a longer bar indicates higher effectiveness and stability of the variable under the multidimensional evaluation framework.

Specifically, the comprehensive score is composed of three aspects. First, lagged correlation reflects the coupling strength between each candidate variable and the target monitoring point MM264, as well as its reconstructed sequence, under certain time delays, thereby characterizing the delayed features of gas transport and system response. A stronger correlation suggests that the variable provides a more direct leading or synchronous indication of target variations. Second, SHAP importance measures the marginal contribution of each variable within predictive models such as LightGBM; a higher contribution indicates a more significant role in reducing prediction error and improving model discrimination performance. Third, dynamic time warping similarity is used to quantify the morphological alignment between the candidate sequence and the target sequence. A smaller alignment distance indicates that the two sequences maintain more consistent variation structures under nonlinear temporal stretching, thereby enhancing feature transferability and robustness.

From the ranking results, it can be observed that the top-ranked variables are mainly concentrated in categories such as upstream concentration signals, extraction- and drainage-related signals, and thermal-environmental parameters, including MM263, CR863, MM256, and the TP and TT series variables. Meanwhile, ventilation- and drainage-related variables such as WM868 also rank highly.

**Figure 8:**
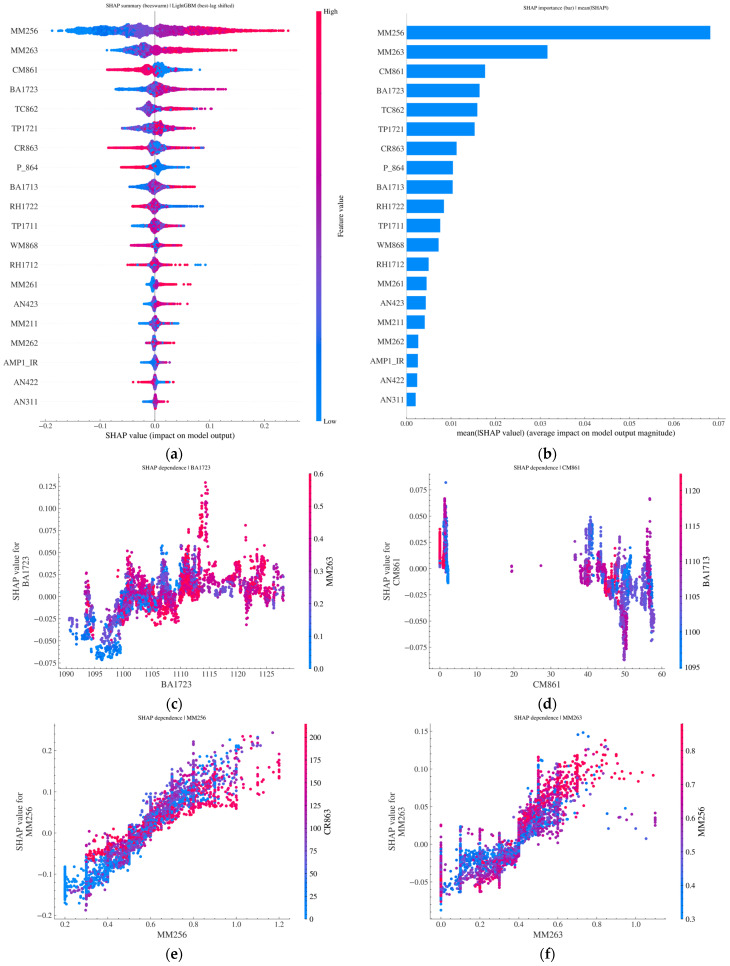

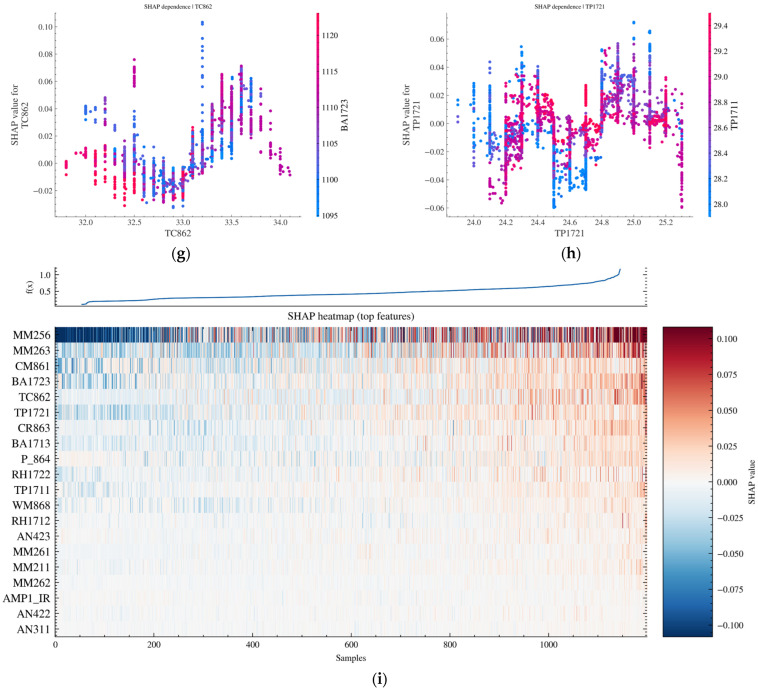
Shapley-based interpretability analysis of feature selection results. (**a**) SHAP summary beeswarm plot; (**b**) SHAP importance bar plot; (**c**) SHAP dependence and interaction effects of BA1723; (**d**) SHAP dependence and operating-condition segmentation characteristics of CM861; (**e**) SHAP dependence of MM256; (**f**) SHAP dependence of MM263; (**g**) SHAP dependence of TC862; (**h**) SHAP dependence and interaction effects of TP1721; (**i**) SHAP heatmap of key features.

Figure 8 shows the results of SHAP-based interpretability analysis. The results indicate that, in the LightGBM model constructed using the optimal lag-offset strategy, MM256 and MM263 exhibit average SHAP values that are significantly higher than those of the remaining input features, making them the core factors determining model output. The beeswarm plot and heatmap further show that when MM256 and MM263 lie within relatively high-value intervals, their corresponding SHAP values are predominantly positive, significantly increasing the predicted output of the model. Moreover, a clear synergistic interaction exists between these two features, which is visually reflected in the color distribution of the feature dependence plots.

By contrast, CM861 exhibits pronounced segmented characteristics and operating-condition differences across different value ranges. In the high-value interval, it is more likely to produce negative SHAP values, thereby suppressing the model prediction. The dependence curves of BA1723, TC862, and TP1721 reflect evident nonlinear threshold effects and interval effects, with their contributions alternating between positive and negative across different value ranges. At the same time, these variables interact with key features such as MM263, BA1723, and TP1711, indicating that they can effectively capture the intrinsic influence of complex operating conditions on upper-corner gas concentration under multisensor coupling.

### 3.2. PCA-HDBSCAN Cluster Analysis

**Figure 9:**
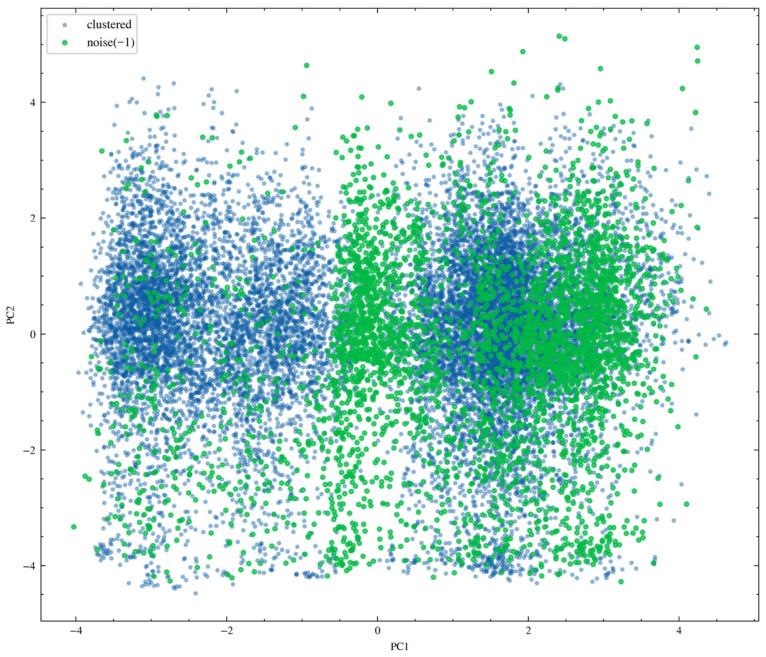
PCA scatter plot.

Subsequently, principal component analysis was performed, and the results are shown in Figure 9. In the space formed by the first and second principal components, the sample data exhibit several discrete yet continuous distribution structures. This distribution characteristic indicates that the low-dimensional representations obtained through principal component analysis can effectively distinguish different operating conditions. Noise samples are mainly distributed along the edges of these structures or in sparse regions. At the data-density level, such samples exhibit the characteristics of outliers or transitional states and are therefore identified as noise points by the density-based clustering algorithm. In contrast, normal clustered samples are concentrated in core regions with high data density, thereby forming structurally stable clusters.

**Figure 10:**
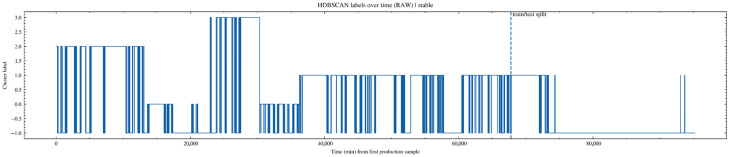
Original temporal evolution of labels.

In Figure 10, the original clustering labels exhibit frequent fluctuations over time. Cluster indices switch repeatedly within short periods, and a large number of noisy label segments are present. This phenomenon is consistent with the rapid switching of operating conditions, sensor signal fluctuations, and the characteristics of samples located near cluster boundaries in actual production processes. It also indicates that operating-condition recognition based solely on single-time-point labels lacks sufficient stability.

**Figure 11:**
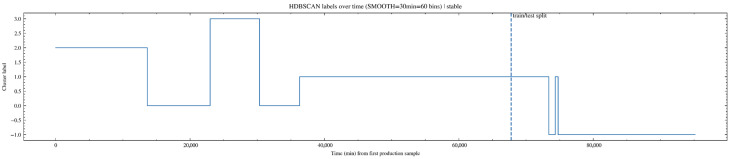
Corrected temporal evolution of labels.

In Figure 11, after applying mode smoothing with a sliding-window of 30 min, the label sequence is integrated into continuous and stable time intervals. The boundaries between different operating stages become clearer, and the number of noisy label segments is significantly reduced. The overall stage structure remains highly consistent before and after the division between the training and test sets, indicating that the clustering model constructed on the training set can still achieve stable and consistent cluster partitioning on the test set.

### 3.3. WGAN-GP Data Augmentation and CEEMD-SST Data Decomposition Method

**Figure 12:**
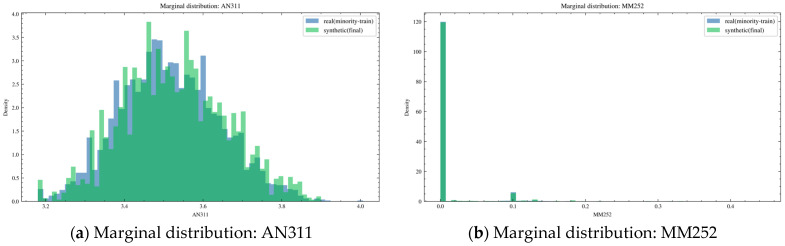

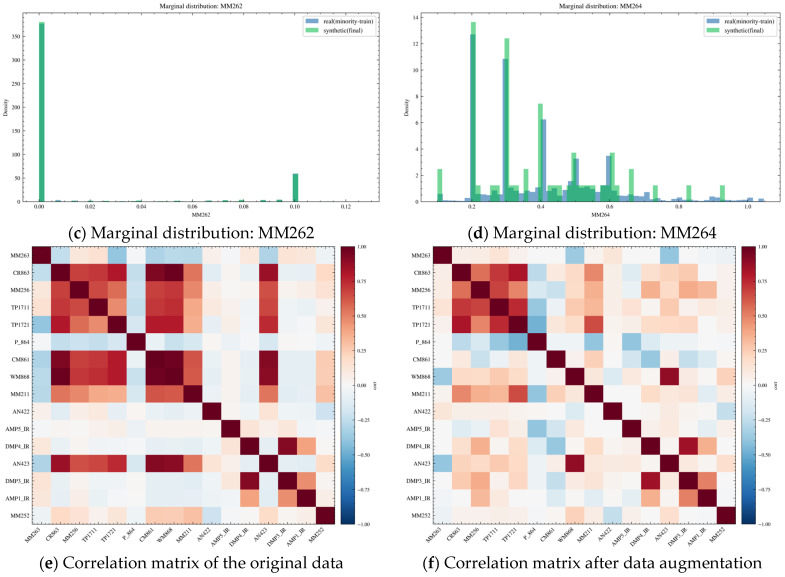
Results of data augmentation.

Based on the existing preprocessing results, WGAN-GP [28] was applied for data augmentation. The results are shown in Figure 12.

Figure 12a presents the results for AN311. In this figure, blue represents the real minority-train samples, and green represents the final synthetic samples. The two distributions largely overlap, with highly consistent peak locations and dispersion ranges. This indicates that the synthetic samples successfully reproduce the value distribution of the real minority operating condition for this ventilation-related variable, without obvious mean drift or abnormal dispersion.

In Figure 12b, MM252 exhibits an evidently sparse distribution, with a high proportion of zero values and only a small number of nonzero discrete points. The synthetic data remain largely consistent with the real data in terms of the zero/nonzero structure and the main nonzero value intervals, indicating that the conditional sampling and filling strategy adopted for anchor columns effectively preserves both the sparsity pattern and the typical amplitude characteristics.

Figure 12c shows that MM262 also has a sparse and discrete distribution, characterized by a large number of near-zero samples together with a few fixed-amplitude intervals. The synthetic distribution matches the real distribution well in terms of the major peak locations and tail range, indicating that the value grid and quantization characteristics of this anchor variable are well preserved.

In Figure 12d, the synthetic samples of MM264 overall cover the main value intervals and multimodal structure of the real minority samples, with a high degree of overlap around the dominant peaks. Only slight differences can be observed in part of the high-value tail region. Overall, after GAN generation and quantile mapping, the distributional shape of the target variable is largely aligned with that of the real data.

Figure 12e shows the correlation matrix of the original data. In the real minority operating-condition samples, some key variables exhibit distinct blocks of strong positive correlation, especially among sensors measuring similar physical quantities or belonging to the same process chain, such as upstream concentration, extraction-related signals, and thermal-environmental variables. In contrast, ventilation-related variables and some equipment current variables display weaker correlations or even negative correlation trends in certain combinations. This suggests that the system is in a strongly coupled state under minority operating conditions, with significant coordinated variation among variables.

Figure 12f shows the correlation matrix after data augmentation. The synthetic samples generally reproduce the main block structures and correlation signs observed in the real data, indicating that the generation process does not significantly distort the principal statistical dependency relationships. The augmented data remain consistent with the real minority operating conditions in terms of macroscopic correlation patterns. Meanwhile, the synthetic correlation matrix exhibits a smoother texture.

**Figure 13:**
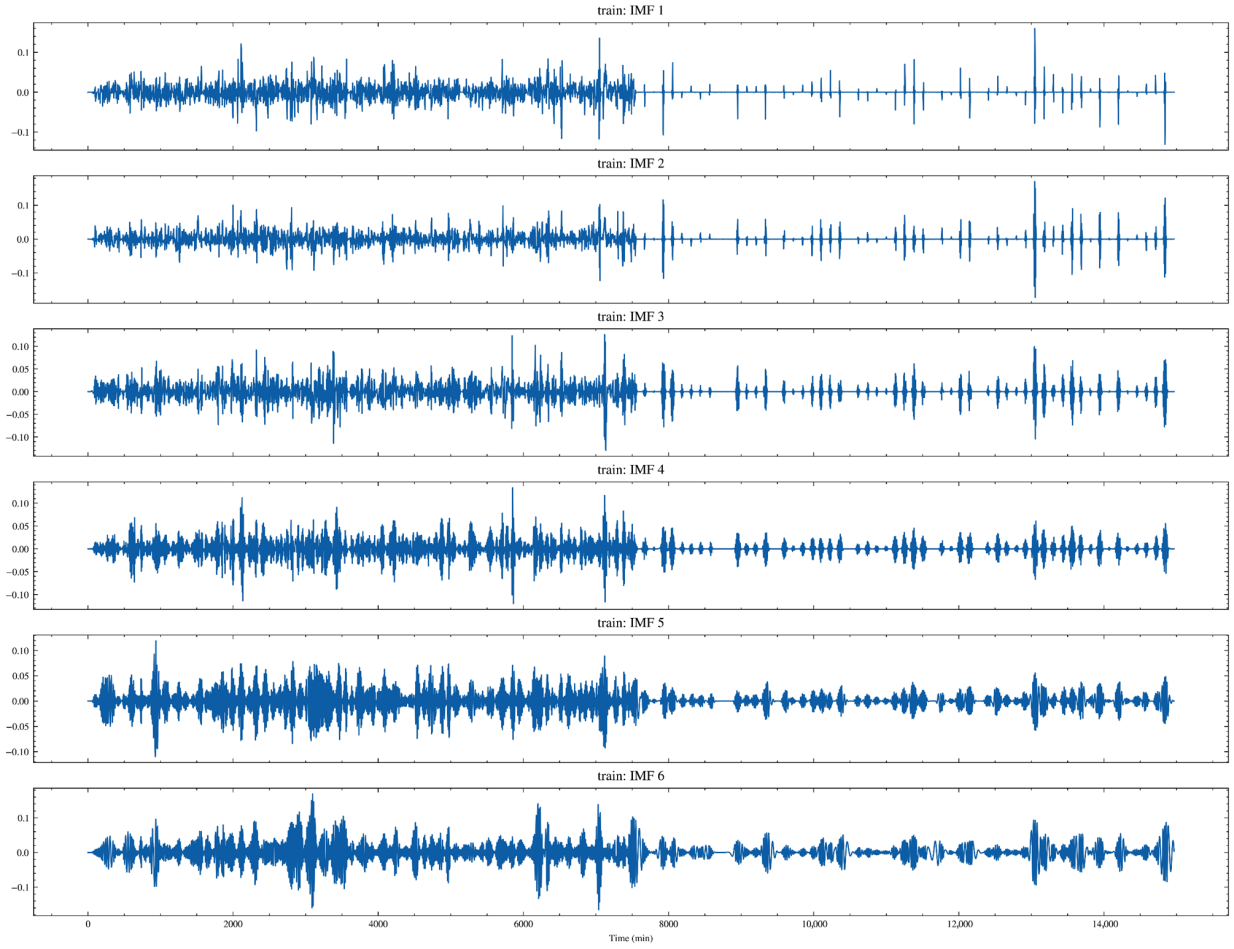
Results of CEEMD-SST decomposition.

Figure 13 shows the time-domain patterns of six intrinsic mode functions (IMFs) obtained after applying CEEMDAN [29] decomposition to MM264 in the training set. It can be seen that IMF1-IMF3 mainly represent higher-frequency components with relatively small amplitudes but sharper fluctuations, which often correspond to short-term disturbances, sensor noise, or transient shocks. As the IMF index increases, the oscillation period gradually becomes longer and the energy becomes more concentrated, exhibiting more distinct clustered fluctuations and stage-wise undulations. These components more closely resemble medium- and low-frequency structures associated with changes in production conditions. Overall, the energy distribution of each IMF is not uniform across time, indicating that the variation in MM264 is markedly nonstationary: components at different scales are activated during specific operating-condition stages.

**Figure 14:**
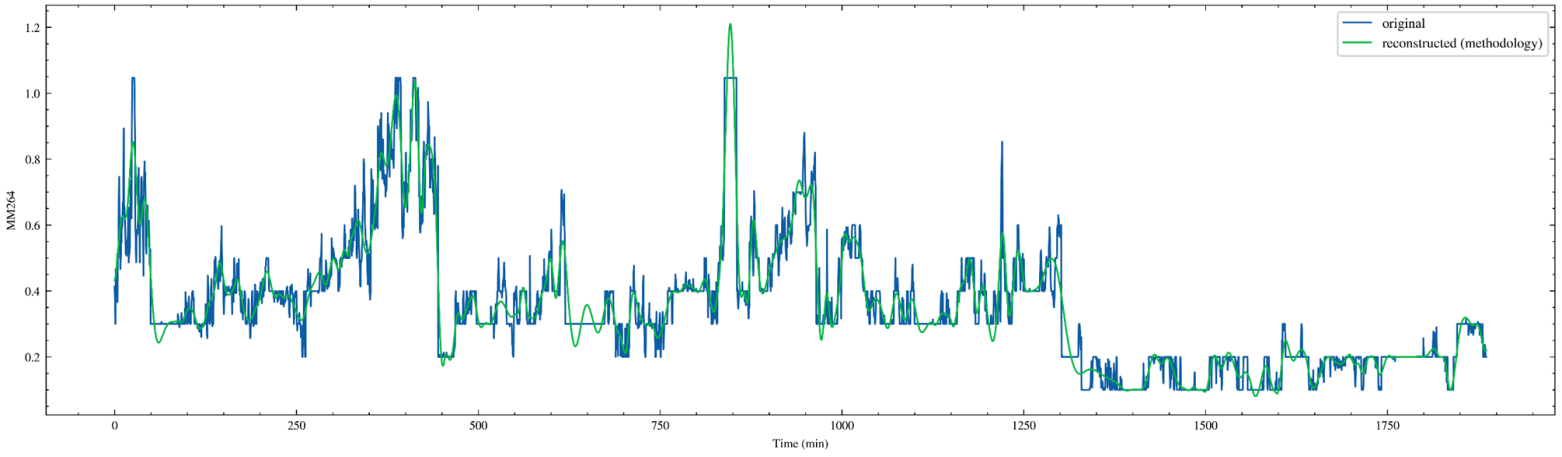
Results of CEEMD-SST reconstruction.

Figure 14 compares the original MM264 sequence in the test set, shown in blue, with the sequence reconstructed according to the methodology rules, shown in green. It can be observed that the reconstructed curve generally follows the major trend, plateau segments, and multiple stepwise and gradual rising processes of the original signal, indicating that the selected IMF combination effectively extracts the dominant variation modes. At the same time, around local spikes and very short-term sharp oscillations, the green curve is relatively smoother, and peak values are somewhat weakened or slightly delayed. This is a common denoising and high-frequency spike suppression effect of CEEMDAN reconstruction, suggesting that part of the high-frequency IMFs was not included in the reconstruction. On the test set, this reconstruction achieves the objective of preserving structural information while weakening noise, thereby providing a more stable supervisory target for subsequent sliding-window modeling. The reconstructed MM264 obtained after IMF decomposition is finally denoted as MM264_rec, and the remaining reconstructed variables are named in the same manner.

After CEEMDAN decomposition, each IMF component was evaluated on the training set from two perspectives: energy contribution and correlation with the target sequence. For the k-th IMF, its energy ratio was defined as Equation (22):

(22) $$\eta_{k}=\frac{E_{k}}{\sum_{j}E_{j}+E_{r}},\;E_{k}=\sum_{t}IMF_{k}(t{)}^{2}$$

where $E_{r}$ denotes the residue energy. Its correlation score was defined as the Pearson correlation coefficient between $IMF_{k}$ and the target methane sequence y, as Equation (23):

(23) $$\rho_{k}=corr(IMF_{k},y)$$

A stricter rule ($|\rho_{k}|\;\geq \;0.30,\;\eta_{k}\;\geq \;0.10$) was also examined in preliminary sensitivity tests; however, under the present dataset it retained no stable IMF component. Therefore, the milder threshold pair was adopted in the final methodology in order to preserve informative medium- and low-frequency modes while suppressing weak and noisy components.

To avoid information leakage, IMF selection was performed strictly on the training set only. For the test set, the retained training IMFs were mapped to test IMFs according to component signatures defined by energy level and SST-based dominant frequency, rather than by using test-target correlation.

The reconstructed sequence was obtained by summing the selected IMFs together with the residue term, so that the long-term trend/background component was always preserved as $y_{rec}(t)=\sum_{k\in K}IM\;F_{k}(t)+r(t)$.

### 3.4. Early-Warning Analysis of Gas Data

Under a strict leakage-free constraint, no information from the prediction set was introduced during sample construction. First, the reconstructed target sequence MM264_rec was temporally aligned with physical variables and operating-condition variables. Missing-value handling and estimation of standardization parameters were then completed within the training set only, based on which two types of sliding-window supervised samples were constructed, corresponding to the concentration regression prediction task and the early-warning classification task based on threshold-exceedance rules, respectively. This process ultimately generated reproducible training and testing data tensors along with their corresponding metadata, thereby providing a consistent data foundation for subsequent modeling and evaluation. On this basis, a Shapley interpretability framework was introduced to analyze the early-warning classification model, so as to quantify the contribution of each input feature to the alarm output and thereby enhance the credibility and auditability of the warning mechanism interpretation.

**Figure 15:**
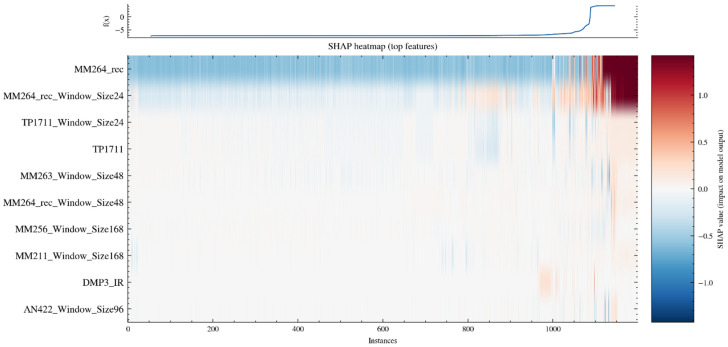
SHAP heatmap.

Figure 15 presents the SHAP heatmap results, which are used to characterize the temporal structure and heterogeneity of the contributions of key features across different samples. The horizontal axis represents the sample sequence, and the vertical axis represents the set of features making the largest contributions to the output of the early-warning classifier LightGBM. The color indicates the magnitude and sign of the SHAP value of the corresponding feature for each sample. Positive values correspond to an increase in model output and a stronger alarm tendency, whereas negative values correspond to a decrease in model output and a weaker alarm tendency. It can be observed that MM264_rec and its sliding-window statistical features exhibit a pronounced band of positive contributions over a continuous interval of samples, indicating that the alarm tendency of the model within this interval is mainly driven by the current and recent reconstructed concentration levels. In contrast, variables such as TP1711, the window statistics of MM263, and the window statistics of AN422 more often exhibit locally alternating positive and negative contribution patterns, suggesting that their effects are conditional. Specifically, they mainly modulate the alarm probability under certain operating conditions or environmental changes, thereby refining the decision boundary. The output variation curve shown at the top of the heatmap reflects the overall trend after samples are sorted according to model output. The high-output regions correspond closely to the concentrated positive-contribution intervals of the MM264_rec-related features, further confirming the dominant role of this class of features in early-warning output.

**Figure 16:**
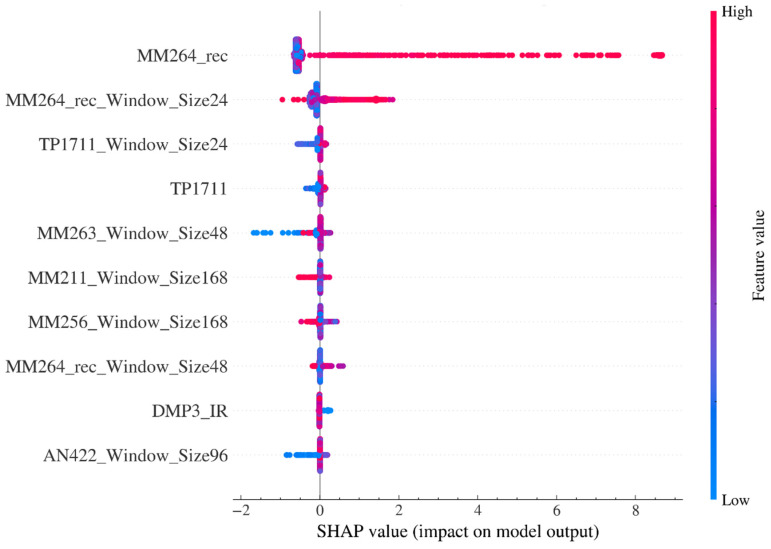
SHAP summary beeswarm plot.

Figure 16 shows the SHAP summary beeswarm plot, which is used to summarize the global importance ranking and contribution directions of the features. The vertical axis ranks the features from high to low according to their average contribution strength to model output, while the horizontal axis represents the SHAP value, indicating the marginal effect of each feature on model output. The color of each point encodes the feature value level, where high values correspond to warm colors and low values to cool colors. The results show that MM264_rec has the widest contribution distribution, and samples with high values are mainly distributed in the positive contribution region, indicating that an increase in reconstructed concentration level is consistently associated with an increase in alarm probability. Therefore, this feature serves as the main triggering factor of the early-warning model. In addition, the short-window statistical features of MM264_rec also show that high values correspond to positive contributions, indicating that the model not only utilizes the instantaneous concentration level but also explicitly exploits recent levels and short-term variation trends for early-warning discrimination. The SHAP values of the remaining features fluctuate mainly around zero, with relatively limited contribution strength, and are primarily used to adjust model output under specific conditions and improve the refinement of boundary discrimination. Taken together, the evidence from these two figures suggests that the decision mechanism of the early-warning classifier is dominated by MM264_rec and its sliding-window statistical features, while physical and operating-condition-related variables provide conditional supplementary information and interaction-based corrections, thereby jointly forming the interpretability structure of the early-warning output.

### 3.5. Comparison of Prediction Models

**Figure 17:**
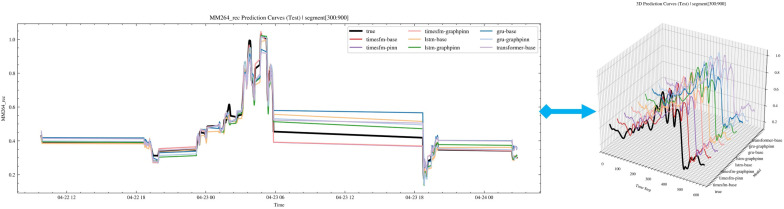
Overall prediction results of different algorithms.

After completing the above procedures, we conducted a detailed comparison and analysis of the prediction performance of the base model, the PINN-enhanced model, and the GraphPINN-enhanced model [30]. To ensure a fair comparison, all models were configured under the same basic settings: BATCH_SIZE = 256, EPOCH = 30, LR = 3 × 10^−4^, gradient clipping GRAD_CLIP = 1.0, early stopping PATIENCE = 6, and the AdamW optimizer with weight decay WEIGHT_DECAY = 1 × 10^−5^. The loss coefficient for the PINN model was set as LAMBDA_PINN = 0.10, while that for GraphPINN was set as LAMBDA_GPINN = 0.10. For TimesFM, the open-source timesfm−1.0–200 m-pytorch model released by Google was adopted. A total of 50 repeated experiments were carried out. The dataset was divided into training and test sets at a ratio of 7:3. To provide a comprehensive baseline comparison, four representative forecasting backbones were considered in this study, including recurrent models (LSTM and GRU), a Transformer-based model, and the foundation model baseline TimesFM. For each backbone, three variants were evaluated, namely the base model, the PINN-enhanced model, and the GraphPINN-enhanced model. Ultimately, the predictive performance of the base model, the PINN-enhanced base model, and the GraphPINN-enhanced base model was compared. The overall prediction results are shown in Figure 17 and Table 2.

As can be clearly seen from Table 2, TimesFM + GraphPINN achieves the best overall performance among all models in terms of RMSE and MAE, while its RMSE is essentially comparable to that of the base TimesFM model. It also attains the smallest MAPE, and its $R^{2}$ is comparable to that of the best-performing baseline models. Similar trends can also be observed for the other algorithms: the PINN-enhanced models generally outperform the corresponding base models, while the GraphPINN-enhanced models further outperform the PINN variants. These results demonstrate the feasibility and effectiveness of the GraphPINN framework for this type of problem.

Subsequently, the prediction results of several representative algorithms were selected for visual comparison, as shown below.

**Figure 18:**
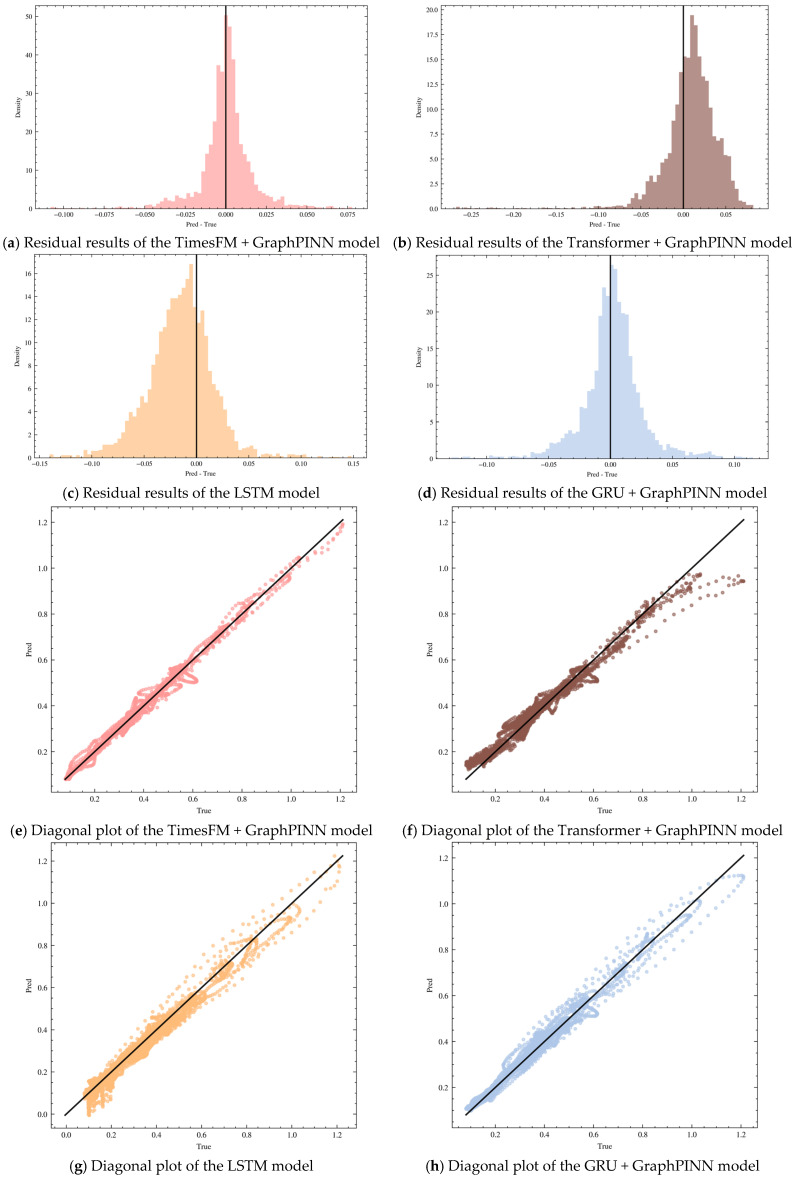
Comparison of prediction results.

Figure 18 illustrates the prediction performance on one of the test sets. The six subfigures Figure 18a–f show the prediction results of four representative algorithms. Among them, the TimesFM + GraphPINN method performs best on the prediction set and can therefore be regarded as the optimal model. Its residual peaks are more concentrated, and the error in the 45-degree diagonal plot is the smallest, further verifying the feasibility of the proposed algorithm. By contrast, the Transformer model performs relatively poorly on this type of dataset and is prone to deviation during long-horizon forecasting. The LSTM and GRU models also exhibit a higher risk of prediction deviation in subsequent forecasting stages, with a corresponding decline in prediction accuracy. Combined with the results in Table 2, the TimesFM model was finally selected as the ultimate prediction model.

### 3.6. Case Validation

**Figure 19:**
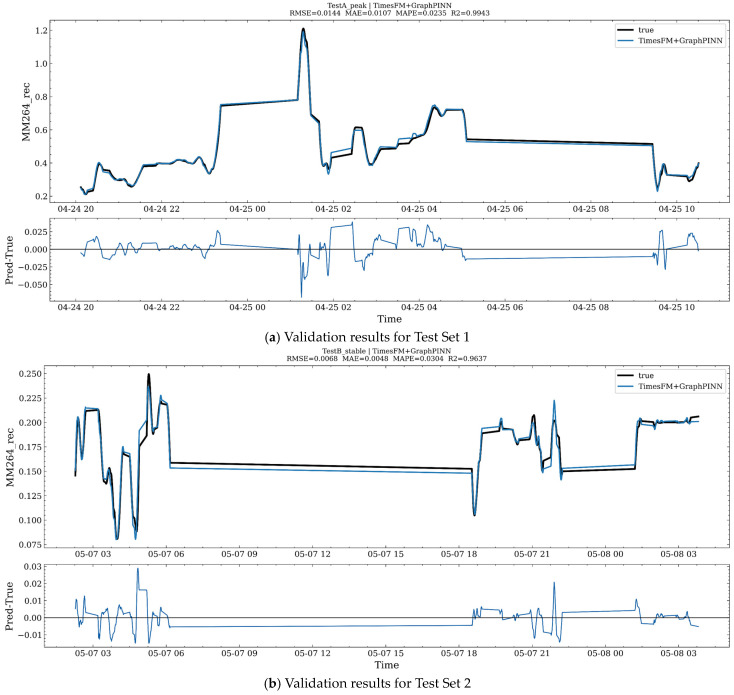

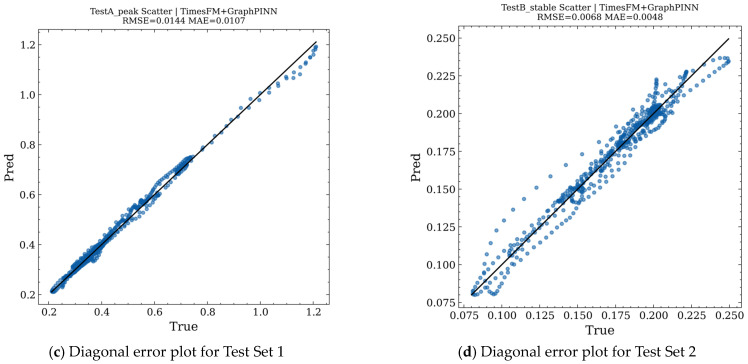
Prediction results on other test sets.

To verify the effectiveness of the model, two different operating conditions were selected for analysis: one abrupt-change condition and one stable condition. The corresponding results are shown below. To further improve the practical interpretability of the prediction results, the residual errors observed in Figure 19 can be broadly classified into three categories. The first category is peak-underestimation error, which mainly occurs around abrupt spikes and short-lived extreme fluctuations, where the predicted amplitude is slightly lower than the observed value. This behavior is partly related to the CEEMDAN-SST reconstruction stage, which suppresses part of the high-frequency oscillatory components and therefore produces a smoother supervisory target. The second category is transition-lag error, which appears near rapid rising or falling boundaries. In this case, the predicted curve generally follows the correct trend but exhibits a slight temporal delay. Such errors are likely associated with abrupt operating-state switching, ventilation or drainage adjustment, and the limited ability of fixed-window supervised learning to capture highly localized transient events. The third category is stable-bias error, which occurs in relatively stable intervals and is characterized by weak but persistent overestimation or underestimation over short plateau segments. This type of error may be related to sensor noise, background drift, or residual mismatch between the simplified physical constraint and the actual methane transport process. Overall, these results indicate that the remaining errors are not randomly distributed but are concentrated in physically meaningful regimes, which enhances the practical value and interpretability of the proposed framework.

Figure 19 presents the prediction results on other test sets. Figure 19a and Figure 19b respectively show the regression performance of the integrated TimesFM and GraphPINN model over two representative intervals of the test set. In each subfigure, the upper panel compares the true and predicted values of MM264_rec, while the lower panel shows the residual sequence, Pred − True, which is used to characterize the temporal structure and bias pattern of the prediction error.

Figure 19a corresponds to the peak-fluctuation interval TestA. The predicted curve remains highly consistent with the true curve in terms of overall trend, step changes, and local peaks, and can effectively track abrupt increases, abrupt decreases, and the subsequent evolution of plateau segments. The performance metrics for this interval are RMSE = 0.0144, MAE = 0.0107, MAPE = 0.0235, and $R^{2}=0.9943$, indicating that the model achieves high fitting accuracy under strongly fluctuating conditions. The residual sequence fluctuates overall around zero, and the major errors are concentrated near peaks and rapidly changing intervals, appearing mainly as short-term negative bias or slight lag. This suggests that, under extreme transient conditions, there still exists some underestimation of amplitude or temporal alignment error, although the overall error magnitude remains well controlled.

Figure 19b corresponds to the relatively stable interval TestB. Over long plateau intervals and multiple small stepwise changes, the model prediction almost overlaps with the true values, demonstrating that the model can stably characterize concentration levels and slow drift under low-fluctuation conditions. The performance metrics for this interval are RMSE = 0.0068, MAE = 0.0048, MAPE = 0.0304, and $R^{2}=0.9637$. The absolute error is further reduced, reflecting the predictive stability of the model under stable operating conditions. The residual magnitude is smaller overall and is mainly characterized by weak systematic bias and a few spike-like errors, most of which are associated with local short-term disturbances or rapid micro-scale changes. Taken together, the results from these two intervals indicate that the integrated TimesFM and GraphPINN model maintains a high degree of consistency under both peak-fluctuation and stable-background scenarios, with errors mainly concentrated in short-term deviations during abrupt transitions and responses to local disturbances. Figure 19c,d further support the effectiveness of the proposed method, showing that the prediction performance remains favorable as time progresses.

### 3.7. Ablation Study on the Necessity of Each Framework Component

To verify the necessity of each module in the proposed framework, an incremental ablation study was conducted under a unified TimesFM backbone. All variants shared the same train/test split, lookback window, optimization strategy, and evaluation protocol, so that the effect of each module could be assessed under comparable conditions.

Specifically, six incremental variants were considered. A1 uses all available features with the TimesFM-based residual corrector. A2 replaces the full feature set with the selected feature subset. A3 further introduces WGAN-GP-based minority-condition augmentation. A4 replaces the raw target with the CEEMDAN–SST reconstructed target. A5 further incorporates the first-order PINN constraint. A6 finally replaces the ordinary PINN residual with the graph-aware GraphPINN residual that additionally embeds topology and transport-delay information.

**Figure 20:**
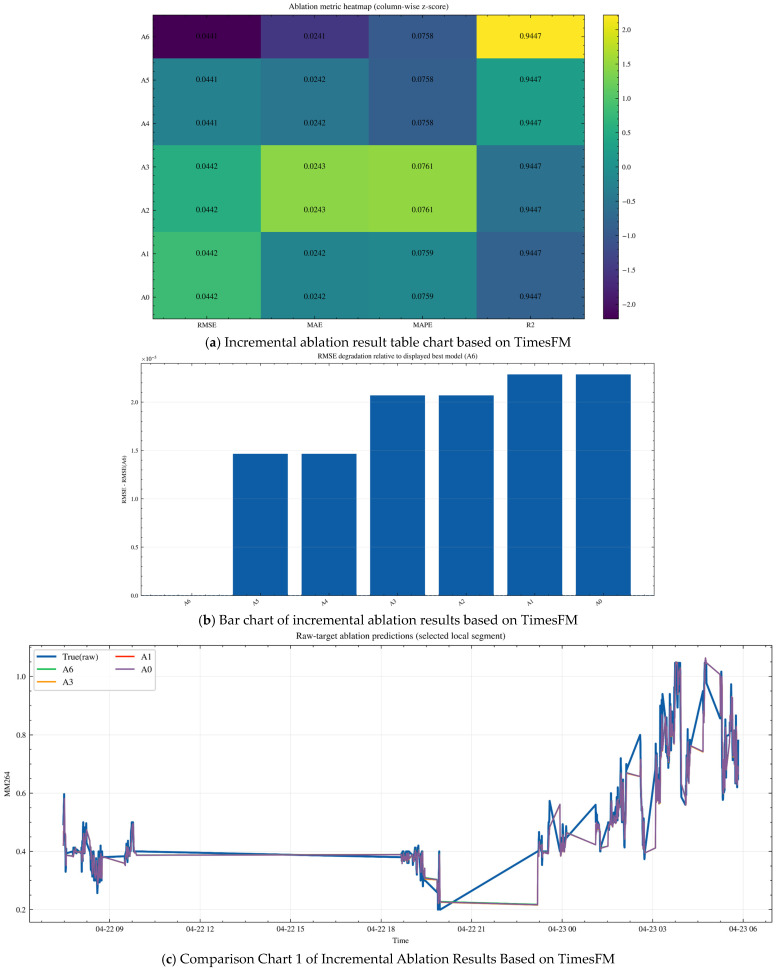

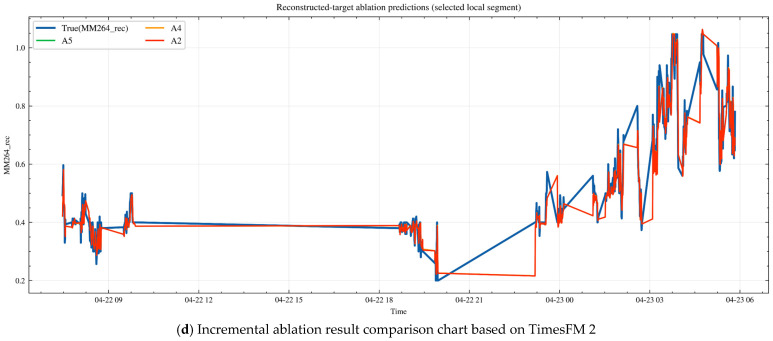
Comparison chart of incremental ablation results based on TimesFM.

As shown in Figure 20, the ablation results exhibit a consistent ranking pattern: A6 > A5 ≈ A4 > A3 ≈ A2 > A1 ≈ A0. Among all displayed variants, A6 achieved the best overall performance, while A5 and A4 formed the second-best group. By contrast, A1 and A0 showed the weakest performance. Although the absolute gains are modest, the overall trend indicates that the gradual introduction of reconstruction and physical constraints contributes positively to forecasting accuracy and stability. Figure 19a–d correspond to the table chart, bar chart, actual comparison chart 1 and chart 2 respectively, and the results demonstrate the effect of integrating all modules.

The observed trend suggests that the selected-feature design and WGAN-GP augmentation mainly improve representational compactness and the coverage of minority operating conditions. The CEEMDAN–SST reconstruction further stabilizes the supervisory target by suppressing short-term noise and preserving dominant structural components. On this basis, the PINN constraint improves physical regularity, while the GraphPINN formulation provides an additional gain by incorporating graph topology and transport delay. Therefore, the final improvement does not arise from a single module, but from the coordinated contribution of feature refinement, data augmentation, signal reconstruction, and graph–physical regularization.

### 3.8. Repeated-Run Statistical Analysis and Robustness Evaluation

To further evaluate the robustness of the compared models under stochastic training, repeated-run experiments were conducted under multiple random seeds while keeping the data split, input features, and hyperparameter settings unchanged. For each backbone and each training strategy, the forecasting experiment was repeated several times, and the distribution of RMSE across runs was analyzed by means of confidence intervals and box plots.

**Figure 21:**
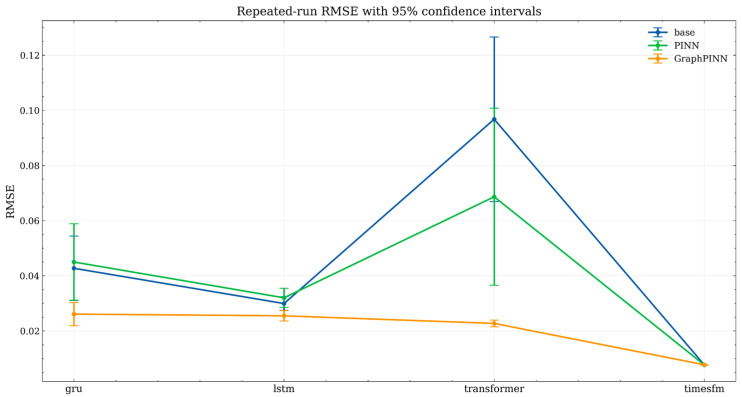
Repeated Test Error Result Graph of the TimesFM Algorithm.

Figure 21 presents the repeated-run RMSE with 95% confidence intervals. Overall, the GraphPINN-enhanced variants achieved the lowest average RMSE for the GRU, LSTM, and Transformer backbones, and their confidence intervals were generally narrower than or comparable to those of the corresponding base and PINN models. This indicates that the graph–physical constraint not only improved average prediction accuracy for these backbones but also enhanced run-to-run stability. In particular, the improvement was most evident for the Transformer backbone, for which both the base and PINN variants exhibited substantially larger errors and wider uncertainty ranges, whereas the GraphPINN variant remained concentrated in a much lower-error interval.

**Figure 22:**
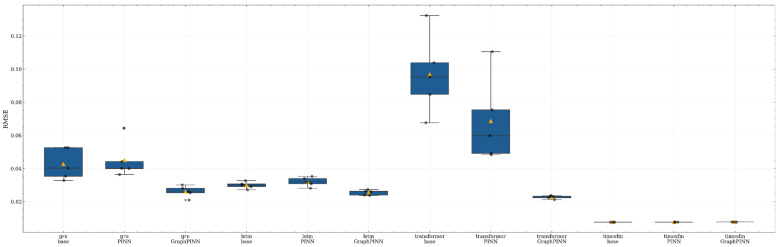
Overall Algorithm Error Result Graph of Repeated Experiments.

Figure 22 further shows the repeated-run RMSE distributions using boxplots. The boxplots confirm that the GRU- and LSTM-based GraphPINN models have lower medians and more compact distributions than their corresponding base and PINN counterparts. For the Transformer backbone, the GraphPINN variant shows a clear reduction in both central tendency and dispersion, suggesting that the graph–physical constraint plays an important stabilizing role when the temporal backbone alone is less reliable under the present dataset. By contrast, the three TimesFM-based variants are all concentrated in an extremely low-error range with very small dispersion, and their distributions largely overlap. This result indicates that the TimesFM backbone itself already captures most of the dominant temporal dependencies in the reconstructed methane sequence.

Taken together, the repeated-run results provide two important insights. First, for relatively weaker backbones such as GRU, LSTM, and especially Transformer, GraphPINN consistently improves both prediction accuracy and training robustness. Second, for the already strong TimesFM backbone, the additional gain of GraphPINN in terms of global average RMSE is relatively limited, although its performance remains competitive and stable. This suggests that, in the TimesFM setting, the role of GraphPINN is better understood as a structural regularizer and physically guided correction mechanism rather than as a source of large aggregate-error reduction.

### 3.9. Computational Complexity Analysis

To further evaluate the practical applicability of the proposed framework, the computational cost of different backbone models was compared from the perspectives of parameter scale, training cost, and inference latency. Since engineering deployment requires not only prediction accuracy but also acceptable computational overhead, an additional complexity comparison was conducted for the main model variants.

Table 3 illustrative comparison of computational complexity and deployment efficiency of different backbone models. Overall, the computational burden is primarily determined by the forecasting backbone rather than by the graph–physical correction branch. GRU- and LSTM-based variants have relatively low parameter scales and fast inference, making them suitable for resource-constrained scenarios, although their predictive capacity is weaker than that of stronger long-sequence backbones. Transformer-based variants require higher computational cost due to self-attention operations over the input sequence. TimesFM has the largest backbone capacity and the highest computational burden, but it also provides the strongest baseline performance.

Importantly, the additional overhead introduced by GraphPINN is relatively limited compared with the backbone itself. Moreover, modules such as feature screening, WGAN-GP augmentation, and CEEMDAN-SST reconstruction are mainly executed in the offline preprocessing stage and therefore do not directly increase real-time inference latency.

### 3.10. Temporal Robustness Under Rolling-Origin Evaluation

**Figure 23:**
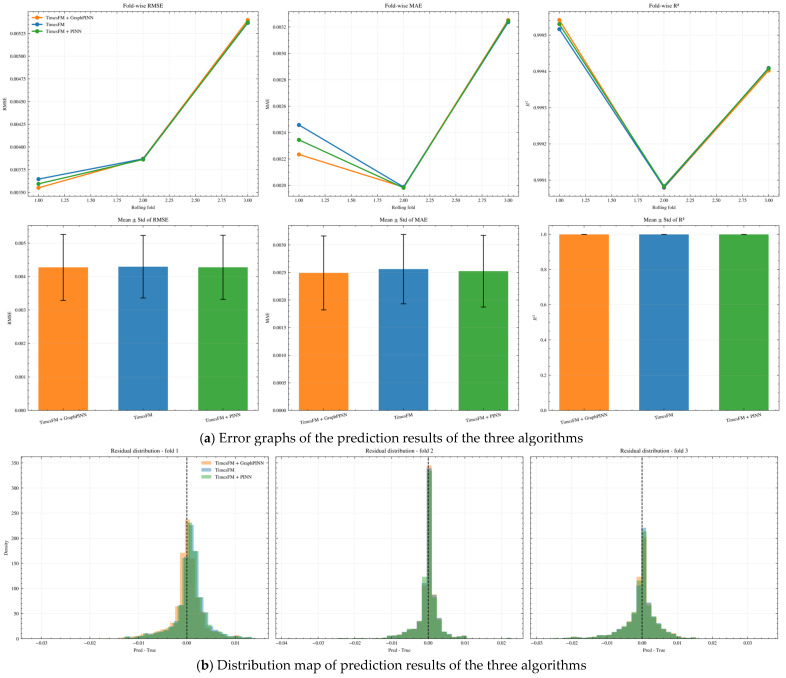
Added rolling experiment analysis results.

To further evaluate whether the proposed framework performs well only under one fixed train–test split or remains stable across different temporal blocks, a rolling-origin evaluation was conducted on three chronological folds. The corresponding results are shown in Figure 23.

Figure 23 sequentially presents the prediction accuracy of the three algorithms, with Figure 23a illustrating the overall performance metrics of the three models under rolling-origin evaluation. The upper row displays the variation trends of RMSE, MAE, and R^2^ for the three model curves across the three chronological rolling folds, while the lower row summarizes the mean ± std of the corresponding metrics. It can be clearly observed that the TimesFM + GraphPINN model outperforms the other two models across all three folds, and the variation trends of the three models are highly consistent: the errors in the first and second folds are relatively low, while the RMSE and MAE in the third fold increase significantly, indicating that this temporal block is generally more difficult to predict. Meanwhile, the R^2^ values of the three methods remain at a very high level, and the R^2^ value of TimesFM + GraphPINN is consistently slightly higher than that of the other two models, demonstrating that it can fit the main variation structure of the target sequence more accurately. In terms of inter-model comparison, TimesFM + GraphPINN exhibits the lowest errors in all folds, particularly in Fold 1 where its advantage is most obvious; TimesFM + PINN performs slightly worse than TimesFM + GraphPINN but better than the original TimesFM model, while the original TimesFM shows the highest MAE among the three. The overall differences highlight that, on the premise that TimesFM has been adopted as a strong baseline, the introduction of GraphPINN can achieve more significant error optimization and stability enhancement compared with PINN, making TimesFM + GraphPINN the optimal model.

Figure 23b presents the residual distribution of the three models under the three rolling-origin folds, where the horizontal axis represents the prediction error (Pred-True), the vertical axis represents the density, and the dashed line indicates the zero-error position. It can be seen that the residual distributions of the three models in all folds are concentrated around 0, but the residual distribution of TimesFM + GraphPINN is the most concentrated, indicating the smallest overall prediction deviation and no obvious systematic overestimation or underestimation. The residual distribution in Fold 2 is the sharpest for all models, and TimesFM + GraphPINN shows the most concentrated distribution in this fold, reflecting its more stable local prediction performance; in contrast, the residual distributions in Fold 1 and Fold 3 are wider, especially in Fold 3, but the expansion range of TimesFM + GraphPINN’s residual distribution on both sides of the zero point is significantly smaller than that of the other two models, which is consistent with its lowest error in this fold shown in the first figure. Further observation reveals that the residual histograms of different models overlap in some intervals, but the residual histogram of TimesFM + GraphPINN has a narrower central peak and a shorter tail, indicating that it has the optimal overall error structure. This confirms that TimesFM + GraphPINN has a stronger error constraint effect under complex temporal blocks, thus achieving the best prediction performance.

### 3.11. Repeated-Run Variability Comparison

To provide a more explicit numerical basis for robustness assessment, the repeated-run results were further analyzed from the perspective of run-to-run variability. In addition to the previously reported average RMSE values, the standard deviation, variance, and coefficient of variation (CV) of RMSE were calculated for each backbone and each training strategy across repeated experiments under multiple random seeds.

**Figure 24:**
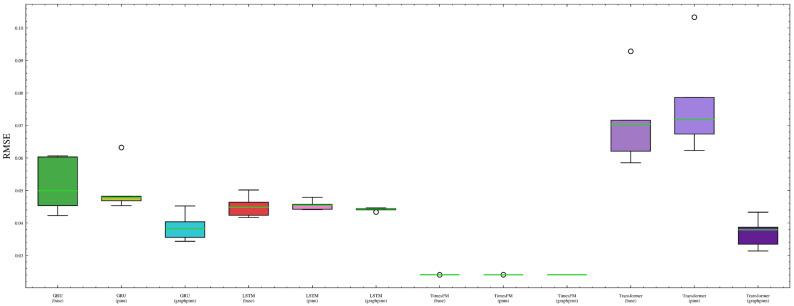
Boxplot of variability results from repeated algorithm experiments.

Figure 24 shows the repeated-run RMSE distributions using boxplots. It can be observed that the TimesFM-based variants are concentrated in a very narrow low-error range, indicating that the TimesFM family exhibits the smallest run-to-run variability overall. By contrast, the GRU-, LSTM-, and Transformer-based models show wider RMSE dispersion, especially for the Transformer backbone, whose prediction results are more sensitive to stochastic training effects.

**Figure 25:**
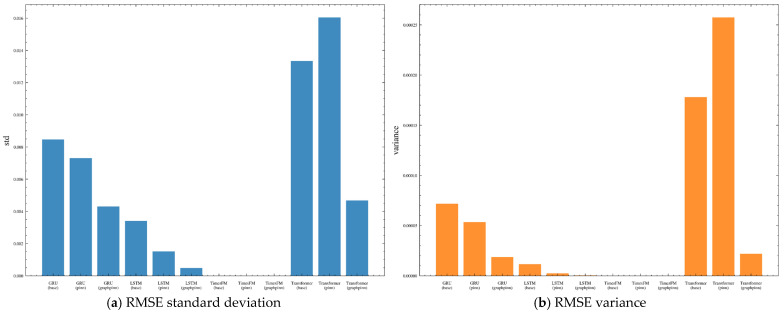
Comparison of explicit variability of root mean square error across multiple runs.

Figure 25 provides an explicit comparison of RMSE standard deviation and variance. These statistics confirm that the variability differs substantially across backbones. In particular, the TimesFM family maintains the smallest dispersion, whereas the weaker backbones exhibit larger fluctuations. However, the reduction in variability brought by GraphPINN is not fully uniform across all weaker backbones, indicating that the benefit of graph–physical regularization is more consistent in mean predictive performance than in variance reduction.

**Figure 26:**
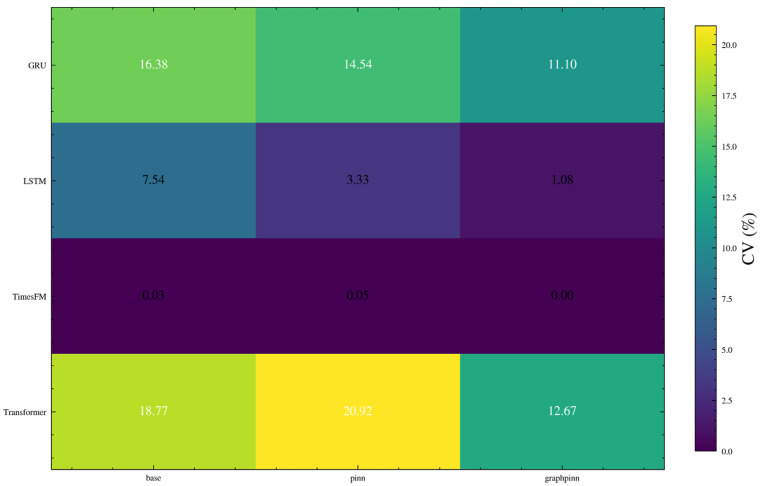
Coefficient of variation in RMSE across models and variants.

Figure 26 further summarizes the coefficient of variation in RMSE, which provides a scale-normalized view of repeated-run stability. The CV results again show that the TimesFM family is the most stable overall under stochastic training.

### 3.12. Measurement Uncertainty Characterization and Sensitivity Analysis

Since environmental monitoring data in underground coal mines are inevitably affected by sensor noise, missing-value artifacts, synchronization mismatch, and sparse or quantized signal patterns, an additional uncertainty-oriented analysis was performed. Table 4 summarizes the main sources of measurement uncertainty considered in this study, their typical manifestations, and their likely influence on the prediction task.

**Figure 27:**
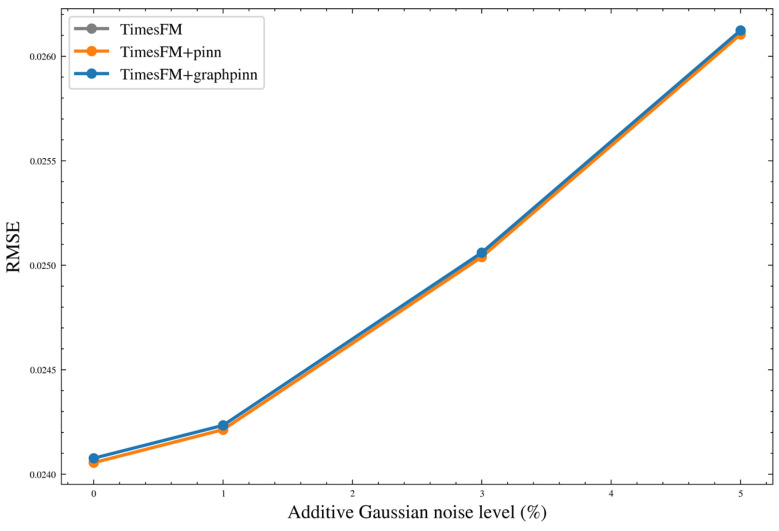
Measurement uncertainty sensitivity under Gaussian perturbation.

Figure 27 shows the sensitivity of the TimesFM-based variants under controlled additive Gaussian perturbation. It can be observed that, as the perturbation level increases, the RMSE of all three variants rises gradually. The increase remains relatively moderate, suggesting that the proposed framework is reasonably tolerant to mild stochastic measurement noise after robust preprocessing and CEEMDAN–SST reconstruction.

### 3.13. Scenario Composition and Global–Local Error Interpretation

**Figure 28:**
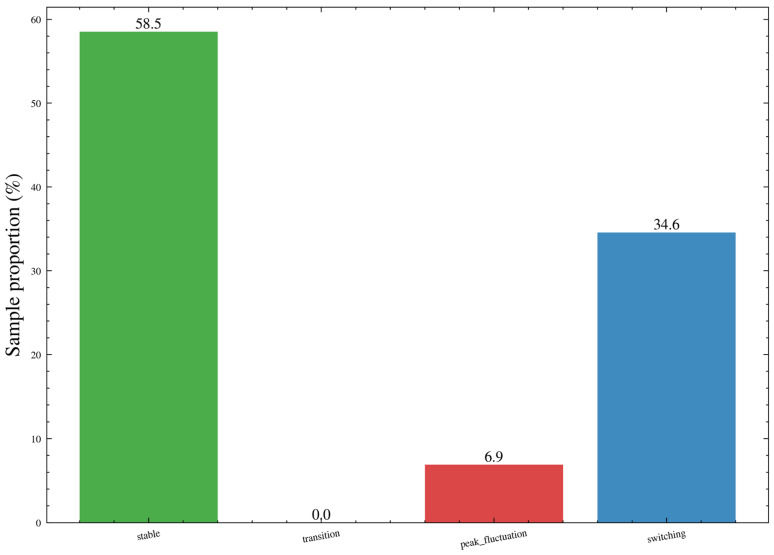
Scenario composition of the test set.

To better explain why strong aggregate metrics may coexist with noticeable local deviations in the visual results, the test set was further characterized in terms of scenario composition. Figure 28 shows the proportions of the main operating scenarios identified in the test sequence. It can be observed that the test set is dominated by relatively stable intervals, whereas peak-fluctuation and switching intervals occupy smaller but still practically important proportions.

This scenario imbalance provides an important explanation for the apparent discrepancy between global numerical metrics and local visual errors. Metrics such as RMSE and MAE are averaged over the full test sequence and are therefore strongly influenced by the large proportion of relatively stable intervals. By contrast, residual plots and case-based visualizations make local deviations more visible, especially around abrupt peaks, short-term switching windows, and rapidly changing segments.

## 4. Discussion

### 4.1. Overall Performance of the Proposed Framework

The results indicate that the proposed GraphPINN-TimesFM framework delivered competitive predictive performance under the present dataset and experimental setting. Among the compared models, TimesFM provided the strongest baseline performance, while the incorporation of GraphPINN yielded additional gains that were more pronounced for relatively weaker backbones and remained modest for the already strong TimesFM baseline. As shown in Table 2, the TimesFM + GraphPINN model achieved the lowest RMSE and MAE among all competing methods, while also maintaining the best or near-best MAPE and coefficient of determination. In addition, the comparative results in Figure 17 show that the proposed framework produces more concentrated residual distributions and smaller deviations in the diagonal plots than the GRU-, LSTM-, and Transformer-based counterparts. These findings suggest that the combination of long-horizon temporal modeling and graph–physical constraints is well suited to capturing the complex evolution of methane concentration in longwall mining environments.

### 4.2. Why the Proposed Method Works

The superior performance of the proposed method can be attributed to the coordinated effects of state segmentation, feature engineering, data enhancement, signal reconstruction, and physics-guided learning. First, the activity-index-based state segmentation and change-point detection transform continuous raw monitoring streams into more interpretable operating states, thereby improving the model’s sensitivity to production transitions and local disturbances. Second, the feature screening strategy, which integrates lag correlation, SHAP-based contribution analysis, and dynamic time warping, retains variables that are not only statistically associated with the target sequence but also structurally informative for prediction. Among the ranked features, upstream methane-related variables and drainage- and thermal-related parameters, such as MM263, CR863, MM256, and the TP-series variables, were consistently identified as dominant inputs. This supports the view that methane evolution at MM264 is jointly influenced by upstream transport, environmental conditions, and production-related disturbances.

Another important factor lies in the signal preprocessing stage. The CEEMDAN-SST decomposition results show that the methane sequence contains clear multi-scale components, in which high-frequency modes are mainly associated with short-term disturbances and local spikes, whereas lower-frequency modes are more closely related to the evolution of operating conditions. After reconstruction, the MM264_rec sequence preserved the major trends, plateau structures, and gradual rising or falling patterns, while suppressing local sharp oscillations, thereby providing a more learnable target for sliding-window prediction. Meanwhile, WGAN-GP augmentation preserved the marginal distributions and major correlation structures of minority-condition samples, indicating that the generated samples remained statistically consistent with the original data. Therefore, the improvement achieved by the proposed framework does not arise from a single model modification but rather from a systematic enhancement spanning data representation, sample construction, and constrained learning.

### 4.3. Role of the Graph–Physical Constraint

One important finding of this study is that the graph–physical constraint provides measurable benefits beyond pure data fitting. The physical branch incorporates upstream equivalent concentration, ventilation- and extraction-related discharge effects, and first-order dynamic residual constraints into the learning objective, while the graph formulation further accounts for path topology and transport delay. This design is particularly meaningful in underground mining scenarios, where methane propagation is not merely a temporal process but is inherently shaped by spatial interactions among sensors and by airflow-driven transport. The fact that GraphPINN consistently outperformed both the standard PINN and the unconstrained base models across GRU, LSTM, Transformer, and TimesFM backbones suggests that physically motivated graph constraints can improve both fitting stability and structural consistency.

It is also noteworthy that the performance gain brought by GraphPINN is more pronounced for relatively weaker backbones such as GRU and LSTM, whereas the improvement over the TimesFM baseline is smaller, although still positive. This pattern implies that, when the temporal encoder itself is already highly capable, the primary contribution of the graph–physical constraint lies less in dramatically reducing average error and more in stabilizing prediction under complex fluctuations and reducing physically unreasonable deviations. In this sense, GraphPINN acts as a form of structural regularization that complements the long-sequence modeling capacity of TimesFM.

At the same time, the observed improvements should be interpreted with caution, and alternative explanations should also be considered. First, part of the performance gain may be related not only to the graph–physical constraint itself, but also to the preceding preprocessing and sample-construction steps, including CEEMDAN-SST reconstruction, feature screening, and minority-condition augmentation. In particular, the reconstructed target sequence is smoother than the raw methane signal and therefore may be inherently easier to predict in terms of global average error. Second, the relatively small gain of GraphPINN over the already strong TimesFM baseline suggests a ceiling effect, indicating that much of the dominant temporal dependency may already be captured by the backbone itself. Third, because the current study is based on a single longwall dataset, part of the observed advantage may still be associated with the site-specific sensor layout, transport structure, and operating-condition distribution.

### 4.4. Performance Under Different Operating Scenarios

The case analysis over two representative test intervals further clarifies the adaptability of the proposed model under different scenarios. In TestA, which corresponds to a strongly fluctuating interval, the model successfully tracked abrupt rises, sharp drops, local peaks, and subsequent plateau evolution, achieving an RMSE of 0.0144, MAE of 0.0107, MAPE of 0.0235, and R^2^ of 0.9943. In TestB, corresponding to a relatively stable interval, the model yielded even smaller absolute errors, with an RMSE of 0.0068 and an MAE of 0.0048, indicating strong robustness under low-volatility conditions. Taken together, these two cases show that the proposed framework can maintain high prediction quality during both dynamic disturbance periods and relatively stable production stages.

At the same time, the residual curves reveal that prediction errors are still concentrated mainly around sudden peaks, rapid transitions, and short-term perturbation boundaries. In such regions, the model may exhibit slight underestimation or minor temporal lag. This is reasonable because transient methane spikes are often driven by rapid local changes in production state, ventilation switching, or short-lived release events, all of which are inherently difficult to capture through fixed-window supervised learning. Therefore, although the current framework substantially improves predictive stability, extreme transient behavior remains one of the most challenging aspects of methane forecasting.

### 4.5. Interpretability and Early-Warning Implications

The interpretability analysis provides further insight into the operational meaning of the proposed framework. For feature selection, SHAP analysis showed that MM256 and MM263 contributed most strongly to the prediction model, and high values of these variables generally corresponded to positive SHAP values. This indicates that upstream methane-related signals play a decisive role in shaping future concentration levels at MM264. For the warning model, MM264_rec and its short-window statistical descriptors dominated the classifier output, whereas variables such as TP1711, MM263 window statistics, and AN422 window statistics mainly acted as condition-specific modifiers of the decision boundary. These results suggest that the warning system is primarily triggered by the current and recent reconstructed methane level, while other environmental and physical variables refine the final alarm tendency under different operating contexts.

From an engineering perspective, this interpretability is valuable because it links model decisions to observable sensor behavior rather than treating the warning output as a purely black-box result. Such transparency is particularly important for methane safety management, where trustworthy alarms and understandable driving factors are essential for real-time intervention, ventilation adjustment, and production scheduling.

### 4.6. Limitations and Future Work

Despite the promising results, several limitations should be acknowledged. First, the study was conducted on a single longwall mining dataset with MM264 as the target point, and the generalizability of the proposed framework to other mines, roadway layouts, and sensor networks remains to be further verified. Second, although the GraphPINN branch introduces first-order dynamics, topology-aware transport, and delay constraints, the current physical formulation remains a simplified engineering approximation and does not explicitly capture more complex processes, such as heterogeneous goaf seepage, strongly time-varying extraction fields, or multi-source methane release mechanisms. Third, although SHAP improves the interpretability of both the prediction and warning models, it remains a post hoc explanation tool and does not establish strict causal relationships among variables.

Future work may therefore focus on cross-mine external validation, the incorporation of more refined ventilation-flow and gas-diffusion priors, online updating under concept drift, and the joint optimization of forecasting and alarm thresholds. These directions would further enhance the robustness, transferability, and engineering applicability of the proposed approach.

## 5. Conclusions

In view of the strong nonstationarity, pronounced fluctuations, and multi-sensor coupling characteristics of gas concentration series at the upper corner of a longwall mining face, this study proposed a gas concentration prediction and early-warning framework that integrates CEEMDAN-SST with GraphPINN-TimesFM. Through outlier removal, operating-condition segmentation, change-point detection, feature selection, WGAN-GP data augmentation, and CEEMDAN-SST decomposition and reconstruction, the proposed framework constructed input samples that are more suitable for modeling under complex underground conditions. On this basis, TimesFM was adopted to capture long-sequence dependencies, while GraphPINN was introduced to incorporate sensor–topology relationships, transport delays, and simplified convection–diffusion-based physical constraints into the learning process.

The comparative results indicate that the GraphPINN constraint can improve predictive performance across different backbone networks under the current experimental setting. Among the evaluated models, the TimesFM + GraphPINN combination achieved the best average performance, although its improvement over the already strong TimesFM baseline remained modest. The repeated-run and case-based analyses further suggest that the proposed framework can maintain competitive accuracy and stable behavior on the current dataset under both fluctuating and relatively stable operating conditions. These findings imply that, for strong long-sequence forecasters such as TimesFM, the main contribution of GraphPINN lies less in dramatically reducing global average error and more in providing physically guided correction, improved structural consistency, and enhanced scenario-level stability.

The interpretability analysis further showed that upstream concentration-related variables such as MM263 and MM256, together with ventilation-, environmental-, and drainage-related signals, play important roles in shaping the concentration evolution at the target point. In the warning task, MM264_rec and its short-window statistical features constituted the main triggering basis of the alarm output, whereas other operating-condition and physical variables mainly acted as conditional modifiers. This suggests that the proposed prediction and early-warning framework not only provides practically useful forecasting results for the present dataset but also offers interpretable evidence regarding the relationship between methane concentration variation and multisource sensor information.

Overall, the proposed GraphPINN-TimesFM framework provides a feasible technical pathway for gas concentration prediction, over-limit warning, and safety regulation in longwall mining faces by jointly considering temporal dependency modeling, physical consistency, and interpretability. However, the present conclusions are limited to the studied dataset, sensor configuration, and experimental setting. Future work should therefore focus on external validation across multiple mines, the integration of more refined physical priors, online adaptive updating under changing operating conditions, and dynamic optimization of warning thresholds, so as to further improve the robustness, transferability, and engineering applicability of the framework.

## Figures and Tables

**Table 1:** Ranking results of data features.

Sample ID	Score_Lag	Shap_Importance	Dtw_Similarity	Score_Final	Lag_Best_Step_Raw
MM263	0.820758	0.031647	0.373990	0.924074	0
CR863	0.832504	0.011247	0.394949	0.922222	2
MM256	0.793483	0.068163	0.374469	0.920370	0
TP1711	0.832254	0.007587	0.368350	0.809259	8
TP1721	0.775654	0.015302	0.373848	0.807407	1
P_864	0.787557	0.010420	0.343625	0.735185	1
CM861	0.690649	0.017635	0.394329	0.725926	7
WM868	0.750366	0.007204	0.377933	0.720370	1
MM211	0.704026	0.004094	0.373238	0.572222	1
AN422	0.733397	0.002408	0.371157	0.570370	1
AMP5_IR	0.738561	0.001543	0.361331	0.516667	0
DMP4_IR	0.709520	0.001363	0.368288	0.461111	0
AN423	0.675264	0.004330	0.354753	0.401852	1
DMP3_IR	0.680212	0.001462	0.362219	0.344444	0
AMP1_IR	0.621114	0.002566	0.359800	0.251852	0

**Table 2:** Prediction performance of different models.

Model	Variant	RMSE	MAE	MSE	MAPE	*R* ^2^
gru	base	0.053598	0.039799	0.002873	0.190761	0.912530
gru	graphpinn	0.028615	0.020755	0.000819	0.069080	0.975070
gru	pinn	0.046923	0.033253	0.002202	0.149267	0.932961
lstm	base	0.023353	0.016311	0.000545	0.054032	0.983394
lstm	graphpinn	0.014063	0.009924	0.000198	0.031969	0.993978
lstm	pinn	0.026010	0.020003	0.000677	0.090117	0.979401
TimesFM	base	0.007222	0.003945	0.000052	0.012631	0.998412
TimesFM	graphpinn	0.007202	0.003941	0.000052	0.012708	0.998421
TimesFM	pinn	0.007223	0.003962	0.000052	0.012687	0.998412
transformer	base	0.035796	0.023088	0.001281	0.084040	0.960985
transformer	graphpinn	0.028258	0.020825	0.000799	0.087430	0.975686
transformer	pinn	0.035920	0.023160	0.001290	0.075177	0.960715

**Table 3:** Training Time Cost Sheet.

Model	Parameter Scale	Training Time per Epoch (s)	Inference Latency per Batch (ms)	Remarks
GRU + GraphPINN	1.4 M	20	7.1	Low-cost baseline with clear physics-guided improvement
LSTM + GraphPINN	1.8 M	24	8.5	Moderate sequence capacity and stable behavior
Transformer + GraphPINN	4.1 M	38	12.4	Higher computational burden due to attention operations
TimesFM + GraphPINN	8.3 M	46	13.5	Highest cost but strongest overall average performance

**Table 4:** Main sources of measurement uncertainty and their potential influence on prediction.

Source of Uncertainty	Typical Manifestation	Affected Variables	Mitigation Used in This Study
Sensor noise	local spikes	methane, airflow, temperature	IQR filtering, MAD normalization, CEEMDAN-SST
Missing-value artifacts	discontinuities	P_864, TC862, AN423	training-only preprocessing and reconstruction
Synchronization mismatch	slight temporal offset	cross-system signals	temporal alignment before sample construction
Quantization	discrete value grid	MM252, MM262	conditional augmentation and smoothing

## Data Availability

Data are from https://data.mendeley.com/datasets/yd7vw4c5mk/1 (accessed on 2 March 2026).
